# Innovative statistical approaches: the use of neural networks reduces the sample size in the splenectomy-MCAO mouse model

**DOI:** 10.3325/cmj.2024.65.122

**Published:** 2024-04

**Authors:** Dominik Romić, Monika Berecki, Sanja Srakočić, Paula Josić Dominović, Helena Justić, Dominik Hamer, Daniela Petrinec, Marina Radmilović, Branimir Hackenberger, Srećko Gajović, Anton Glasnović

**Affiliations:** 1 Department of Neurosurgery, Dubrava University Hospital, Zagreb, Croatia; 2 Croatian Institute for Brain Research, University of Zagreb School of Medicine, Zagreb, Croatia; 3 Department of Biology, “Josip Juraj Strossmayer” University, Osijek, Croatia; * Authors contributed equally.

## Abstract

**Aim:**

To compare the effectiveness of artificial neural network (ANN) and traditional statistical analysis on identical data sets within the splenectomy-middle carotid artery occlusion (MCAO) mouse model.

**Methods:**

Mice were divided into the splenectomized (SPLX) and sham-operated (SPLX-sham) group. A splenectomy was conducted 14 days before middle carotid artery occlusion (MCAO). Magnetic resonance imaging (MRI), bioluminescent imaging, neurological scoring (NS), and histological analysis, were conducted at two, four, seven, and 28 days after MCAO. Frequentist statistical analyses and ANN analysis employing a multi-layer perceptron architecture were performed to assess the probability of discriminating between SPLX and SPLX-sham mice.

**Results:**

Repeated measures ANOVA showed no significant differences in body weight (F (5, 45) = 0.696, *P* = 0.629), NS (F (2.024, 18.218) = 1.032, *P* = 0.377) and brain infarct size on MRI between the SPLX and SPLX-sham groups post-MCAO (F (2, 24) = 0.267, *P* = 0.768). ANN analysis was employed to predict SPLX and SPL-sham classes. The highest accuracy in predicting SPLX class was observed when the model was trained on a data set containing all variables (0.7736 ± 0.0234). For SPL-sham class, the highest accuracy was achieved when it was trained on a data set excluding the variable combination MR contralateral/animal mass/NS (0.9284 ± 0.0366).

**Conclusion:**

This study validated the neuroprotective impact of splenectomy in an MCAO model using ANN for data analysis with a reduced animal sample size, demonstrating the potential for leveraging advanced statistical methods to minimize sample sizes in experimental biomedical research.

In preclinical and clinical research, ethical constraints often limit the number of samples, particularly in experimental studies where rigorous ethical criteria for animal procedures are justified. Efforts are increasingly directed at reducing the number of animals while achieving equivalent results in proving hypotheses (1-3). However, there are limitations to reducing the number of animals, particularly in relation to study power, and the number of subjects needed for experiments using frequentist statistical analysis cannot be significantly reduced. Recently, new data analysis models, particularly neural networks, are being introduced into experimental research, showing great promise by learning from available data and distinguishing between groups with a high degree of certainty without the need for a substantial increase in the number of subjects. By incorporating new data into the existing set of samples, they can, with a high degree of confidence, distinguish between two groups (4-7).

In our laboratory, completing experiments without at least a hinted increase in the number of animals has been challenging, especially in the demanding middle carotid artery occlusion (MCAO) model in mice. Attempting alternative analysis models that provide scientifically equivalent answers on the existing number of animals has been explored. One intervention in the MCAO model, such as prior splenectomy, has shown favorable outcomes in mice (8-11). The spleen, as the largest secondary immune organ in the human body, serves as a significant reservoir of undifferentiated monocytes that mobilize to injury sites after ischemia (12,13). Consequently, splenectomy could potentially affect the availability of circulating monocytes reaching the ischemic lesion, which indicates the spleen's involvement in the immune response during the acute phase following a stroke. Therefore, it could present an appealing target for immune therapies aimed at curbing these harmful reactions (14,15). Several preclinical studies have explored the combination of splenectomy with ischemic stroke models in rodents, yielding varying outcomes. While some studies found that splenectomy before a stroke was associated with a reduced infarct size and improved neurobehavioral outcomes, others failed to show significant benefits from spleen removal (8). However, considering the inherent challenges of the MCAO procedure for animals, adding another surgical intervention increases the chance of animal mortality, raising uncertainties about their number.

Ischemic brain injury initiates an inflammatory response characterized by blood-brain barrier breakdown, allowing peripheral macrophages to infiltrate and activate resident microglia, the central nervous system's principal immune cells. Toll-like receptors (TLRs) are pivotal in this response, recognizing cellular debris and triggering inflammation that influences lesion extent. While targeting TLRs holds therapeutic promise, interventions must carefully navigate the delicate balance between their beneficial and detrimental effects across various stages of ischemic brain injury for effective treatment strategies (16-21).

Based on the existing knowledge, our hypothesis was that splenectomy in mice before MCAO procedure, without necessitating an increase in the animal count during the experiment, would yield favorable neuroprotective outcomes. We aimed to validate this hypothesis through traditional frequentist statistical methods and subsequently employ neural networks on the same data set.

## Materials and methods

The mice groups under study consisted of transgenic B6-Tyrc-Brd-Tg(Tlr2-luc/gfp)/Gaj mice bred at the Croatian Institute for Brain Research, University of Zagreb School of Medicine (approval number HR-POK-006). These mice offer insights into Tlr2 gene expression while functionally resembling wild-type mice (highly inbred C57BL/6 strain). The mice were divided into two experimental groups (totaling 27 animals that survived both procedures): mice that were first splenectomized and then underwent MCAO (SPLX) and mice that were first sham-operated and then underwent MCAO (splenectomy control - SPLX-sham). Splenectomy was performed 14 days before MCAO to allow a reasonable recovery period and biophysical readiness for the MCAO procedure. Ethical approval was granted by the University of Zagreb School of Medicine.

### Splenectomy procedure

The mice were anesthetized by inhalation of isoflurane (Isoflurane, Abbott, Maidenhead, UK; dosages detailed in the MCAO procedure description). The spleen was removed via a dorsolateral approach, the fur around the 13th rib was shaved, the area was disinfected with 70% ethanol, and an incision was made below the 13th rib to access the spleen. It was separated from its blood vessels using electrocautery, and the wound was closed with metal clips. The animals were monitored for one day in a heated cage with analgesia using 0.1 mL of buprenorphine (Buprenovet, Bayer, Leverkusen, Germany). Full recovery from the operation was deemed after a week (22,23).

### MCAO procedure

After measuring body weight, the mice received an intraperitoneal injection of buprenorphine at a dose of 0.05 mg/kg. They were then anesthetized with 3% isoflurane in a closed chamber mixed with 70% N_2_O and 30% O_2_, and the operation was continued with 1.5%-2% isoflurane on a heated pad to maintain body temperature at 37.0 ± 0.5 °C. Fur was shaved in the neck area, disinfected with 70% ethanol, and a central incision was made to access the common carotid artery, carefully separated from the surrounding tissue. The middle cerebral artery was occluded by inserting a silicone-coated surgical filament - 6-0 monofilament thread (Doccol Corporation, Sharon, MA, USA) through the left internal carotid artery to the origin of the middle cerebral artery until resistance was felt (9-10 mm from the bifurcation of the common carotid artery). The silicone-coated monofilament remained in the blood vessels for 60 minutes, then was removed, and the neck wound was closed. Fluid loss during the surgery was compensated by injecting 1.0 mL of saline solution intraperitoneally. Mice recovered on a heated pad for 24 hours post-procedure. Twelve hours after inducing ischemia, mice were again injected with buprenorphine at a dose of 0.05 mg/kg intraperitoneally. Food and water were provided *ad libitum* (24,25).

### Body weight measurement

Mice body weight, post-splenectomy or sham operation, was measured at various times during the study: before surgery, before MCAO, and at two, four, seven, 14, and 28 days after MCAO. Body weight was measured using a precise scale, placing the mice in a plastic container. Weight measurements were recorded in duplicate, averaging the two readings. These measurements were vital to determining the overall health impact of splenectomy or sham operation on mice and if MCAO affected their body weight.

### MRI procedure

To visualize and quantify the lesion, a preclinical MRI system Bruker BioSpec 70/20 USR (Bruker, Billerica, MA, USA) designed for small animal imaging with a 7 T field strength was used. The mice were anesthetized with 2% isoflurane inhalation and placed in a specialized holder allowing head fixation. The holder was heated to prevent the mice from lowering their body temperature during imaging, and body temperature and breathing rate were monitored with sensors. Inhalation anesthesia was adjusted based on sensor data to maintain the mouse in optimal conditions during imaging. Brain imaging was done with a two-channel coil specific to the brain, while spleen imaging used a coil for the entire mouse body. The holder was placed in the device, and depending on the experiment, T1, T2, or DTI sequences were applied. The imaging lasted 30 minutes (26,27). Imaging was conducted in the coronal plane to visualize the entire brain infarct area. The acquired images were analyzed to determine the brain infarct area, measured as an area of increased signal intensity compared with normal brain tissue. Repeat scans were conducted at appropriate time points to monitor stroke progression.

### Bioluminescent imaging (BLI)

Bioluminescence imaging is a non-invasive technique that uses the light emission of specific organisms to visualize biological processes. This method involves the use of transgenic mice expressing luciferase, an enzyme catalyzing the reaction which emits light under the control of a specific promoter. The luciferase substrate is then injected into the mouse, and the emitted light can be detected with a specialized camera. The intensity of the emitted light is proportional to the level of luciferase expression. The maximum increase in bioluminescence refers to the total number of photons emitted by bioluminescent cells or tissue per unit of time, measured in photons per second, representing the overall light quantity emitted by the sample over time. On the other hand, the maximum bioluminescent radiance refers to the amount of light emitted by the sample per unit area, measured in photons per second per square centimeter per steradian (sr), representing the light intensity emitted by the sample at a specific time point.

The mice were anesthetized with 2% isoflurane inhalation and placed in an imaging chamber. They received an intraperitoneal injection of 150 mg/kg D-luciferin (Promega, Madison, WI, USA) just before imaging. The procedure takes about 20 minutes. Imaging (MRI and BLI) were conducted six times – 14 days before MCAO; a day before MCAO; two days after MCAO; four days after MCAO, seven days after MCAO, and 28 days after MCAO.

### Neurological testing

Functional neurological testing was conducted throughout the study to monitor neurological damage after MCAO according to the Garcia method (25). We measured the ability of the mice to perform motor and sensory tasks. Results from each task were recorded and combined to derive an overall neurological score for each mouse. Trained personnel, blinded to each mouse's treatment, performed the assessments. These tests were performed before each procedure and imaging session, namely on the day before the splenectomy procedure, 14 days after splenectomy, immediately before the MCAO procedure, the day after MCAO, just before MRI, and every time before MRI and BLI (28,29).

### Histological analysis

Immunofluorescent analysis of GFAP, TLR2, Iba-2, CD68, and NeuN was conducted on mouse brain tissue to determine the expression levels of these markers. Brain tissue was collected after MRI scanning at specific time points post-MCAO induction. Twenty-eight days after MCAO, anesthetized mice underwent perfusion fixation to isolate the brain for further immersion fixation. After anesthesia with tribromoethanol (Avertin; 0.5 g/kg i.p.), the mice were placed on an operating table, and the abdomen and chest areas were wiped with 70% ethanol. The abdominal and chest cavities were opened with a T-shaped incision, a 22 G needle was inserted into the left ventricle, and the right atrium was cut to enable fluid drainage from the circulatory system. Then, 10 mL of saline solution was injected. Toward the end of perfusion, transparent fluid drained from the right atrium, and the liver appeared pale yellow, which indicated adequate circulatory system flushing. This was followed by perfusion with a 4% paraformaldehyde fixative solution (+4 °C) using moderate pressure (5 mL/min). The brain was extracted from the fixed animal by laminotomy and gentle separation with watchmaker's forceps (30). The tissue was rapidly frozen in isopentane and stored at -80 °C. Tissue sections, 35 μm thick, were cut and mounted on slides. These sections were then stained using primary antibodies specific to GFAP, TLR2, Iba-2, CD68, and NeuN. Primary antibodies, dissolved in a solution of PBS containing 1% goat serum and 0.1% Triton X-100, were applied and left to incubate overnight at room temperature. Subsequently, the sections were incubated for two hours at room temperature with corresponding secondary antibodies, dissolved in PBS. Finally, brain sections were treated with DAPI solution (5 nM, Abcam, USA), covered with fluorescence mounting medium S3023 (Dako, Glostrup, Denmark), and stored at +4 °C. Imaging was performed using a confocal microscope (Olympus Fluorview 3000, Olympus, Tokyo, Japan) equipped with four excitation lasers (405 nm, 488 nm, 561 nm, and 640 nm). Images were captured using a 20 × objective lens (UPlanSApo, Olympus) with a resolution set to 1024 × 1024 pixels. Uniform exposure parameters were maintained for all images.

### Statistical analysis

Frequentist statistics was done first. According to currently available data on a similar model, the number of samples per group for testing the hypothesis of the beneficial effects of splenectomy in the mouse MCAO model is 13. After checking the normality of variable distributions with a Shapiro-Wilk test, analysis of variance (ANOVA) or Kruskal-Wallis H test were performed. The significance level was set at *P* < 0.05.

### Using neural networks to assess hypothesis probability

**Figure 1:**
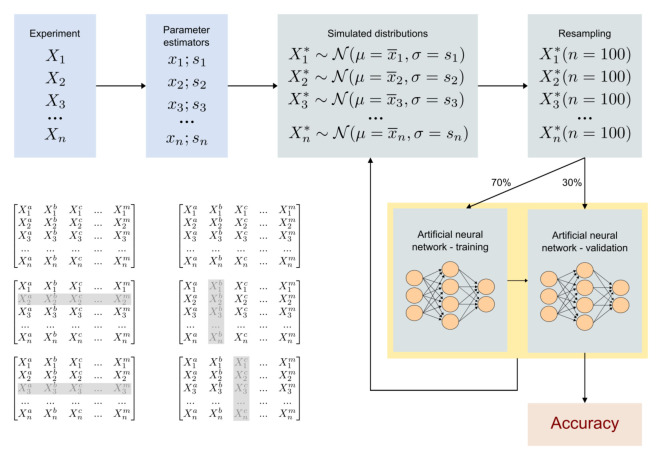
*In silico* experiments for testing hypotheses using simulations with neural networks.

Frequentist statistics' reliance on sample size for reliability poses challenges, as small samples decrease test power and may lead to Type I errors. Considering the principles of frequentist statistics and indications from Bayesian analysis, a neural network was employed to distinguish between SPLX and SPLX-sham mice based on data within a 95% confidence interval. The neural network was trained and validated using artificial populations suggesting that splenectomy had an effect undetectable by common statistical methods (Figure 1).

The data set comprised simulated populations based on parameter estimators. A multi-layer perceptron (MLP) neural network architecture was used, with data randomly divided into 70% for training and 30% for validation. A total of 127 combinations of seven variables were explored, with each neuron in the input layer representing a single variable. Hidden layers utilized rectified linear unit and sigmoid activation functions, with dropout layers to prevent overfitting. The Adam method optimized network performance, adjusting the learning rate dynamically during training. Python with the Keras library was used for implementation.

### Statistical processing of ANN prediction accuracy data

The accuracy of predicting SPLX and SPL-sham classes using ANN was compared for each data set created by excluding data from specific days after stroke (no exclusion, exclusion of Day 2, and exclusion of Days 2 and 4) and variables and their combinations (seven variables, 127 combinations). The normality of data group distributions was tested with a Shapiro-Wilk test (α = 0.05). The existence of a statistically significant difference between the mean values of two data sets was tested with a *t* test (α = 0.05). The mean values of ANN prediction accuracy after excluding certain variables and their combinations were compared with the mean value of ANN prediction accuracy with ANOVA without excluding variables (α = 0.05), and statistically significant differences between groups were determined with a Dunnett's test (α = 0.05). Results are expressed as mean ± standard deviation (SD). All conventional statistical processing was performed with the statistical environment R/RStudio (R.4.3.2/RStudio 2023.12.0) (31). Classification was performed with neural networks in the architecture of an MLP. The training was carried out using a bootstrapping method with the Python 3.9 programming language (32) and the packages Numpy v1.24.0 (33), SciPy v1.10.0 (34), Pandas 2.0.0 (35), Scikit-Learn 1.2.2 (36), Tensorflow 2.1.3 (37), and Keras 2.10.0 (38). The neural networks were trained on a computer with two NVIDIA Quadro GTX 8000 graphics cards (2x48GB VRAM), 128 GB RAM, and an Intel i7-9700K 3.60GHz processor. The artificial neural networks had seven hidden layers with a total of 20 310 parameters and occupied approximately 67 GB of RAM after integration into a consensus-based (39) ensemble and during inference.

## Results

### Initial point as experimental design

**Figure 2:**
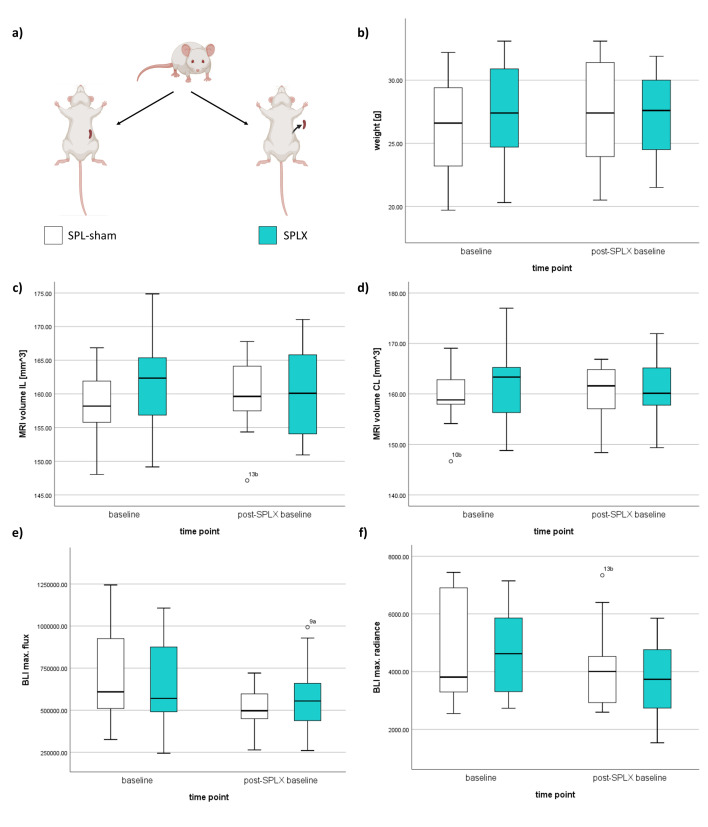
Baseline characteristics of the experimental groups. (**A**) Mice distribution into groups before experimental procedures: splenectomized mice group (SPLX) – gray; sham-operated group (SPL-sham) – white. (**B**) Mean body weight values of mice. (**C**) Mean values of ipsilateral hemisphere (IL) volume at baseline. (**D**) Mean values of the contralateral hemisphere (CL) volume at baseline. (**E**) Mean values of maximum bioluminescence (BLI) increase at baseline. (**F**) Mean values of maximum BLI radiation at baseline.

Animals were randomly divided into two groups. Both underwent the MCAO procedure, with 13 animals previously splenectomized and 14 animals undergoing a sham operation. All sham-operated animals survived MCAO and continued the experiment, while one splenectomized animal died before MCAO. Additionally, two animals underwent splenectomy and later sham MCAO, but they were not included in the experiment, only monitored before other tests. All animals survived for 28 days and were later sacrificed as per protocol. No significant differences in baseline characteristics were observed among animals or between groups (Figure 2).

### Spleen removal before MCAO did not affect mice body weight

**Figure 3:**
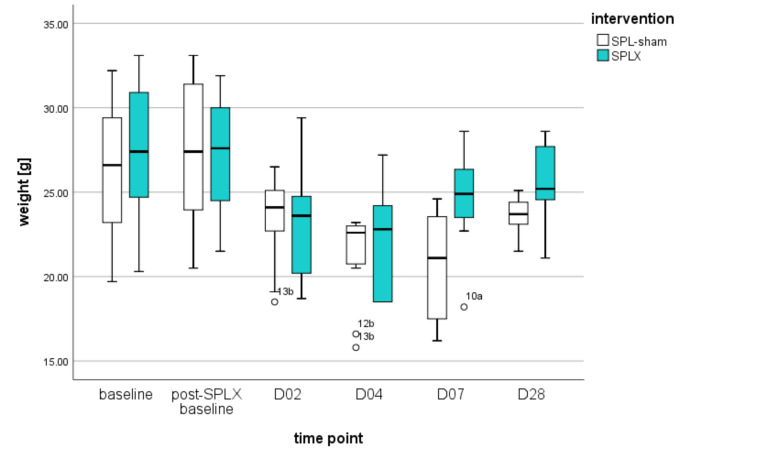
Dynamic changes in mice body weight 14 days before middle cerebral artery occlusion (MCAO), day of the MCAO procedure (D0), and day two, four, seven, and 28 after MCAO. We used *t* test for testing the statistical differences between the groups for every time point. Outliers are also shown in the plot, and each was designated for a better overview. SPL-sham – sham operated mice for splenectomy; SPLX – splenectomized mice.

Results comparing mice body weight before spleen removal and just before MCAO showed that the time period of one week (F (1,18) = 44.960, P < 0.001) but not spleen removal (F (1,18) = 0.070, P = 0.794) significantly influenced the mice weight. Repeated-measures ANOVA indicated no significant difference in body weight between the splenectomized and sham-operated groups (F (5, 45) = 0.696, P = 0.629) after inducing cerebral ischemia. However, a significant difference in body weight was observed in both groups over 28 days post-MCAO compared with baseline (F (5, 45) = 17.901, *P* < 0.001). This suggests that cerebral ischemia affected the mice body weight, but the effect was unrelated to spleen removal (Figure 3).

### Splenectomy did not affect the neurological score of mice after MCAO

**Figure 4:**
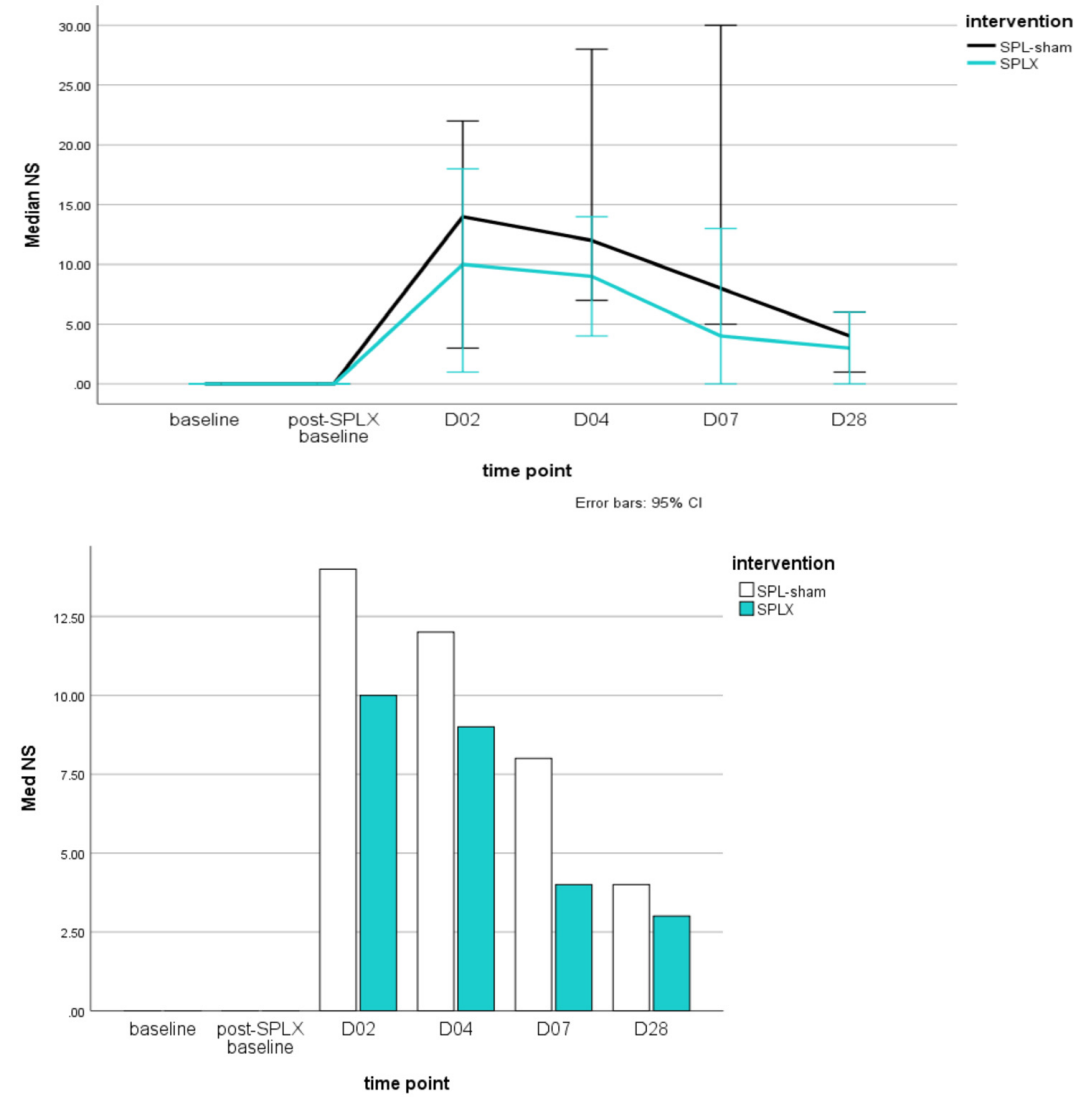
Dynamic changes in mice neurological scores (NS) on day two, four, seven, and 28 after middle cerebral artery occlusion (MCAO). We used *t* test for testing the statistical differences between the groups for every time point. SPL-sham – sham operated mice for splenectomy; SPLX – splenectomized mice.

Neurological assessment results indicated no significant difference between SPLX and SPLX-sham groups post-MCAO (F (2.024, 18.218) = 1.032, *P* = 0.377). However, significant differences in neurological outcomes were observed in both groups 28 days after MCAO compared with baseline (F (2.024, 18.218) = 15.24, *P* < 0.001). This suggests that neither splenectomy nor sham operation significantly affected the overall neurological function of mice, but MCAO notably affected their neurological outcomes. Further analysis is needed to determine the precise cause of the observed neurological changes in both groups (Figure 4).

### Splenectomy did not affect the MRI brain infarct volume in mice subjected to MCAO

**Figure 5:**
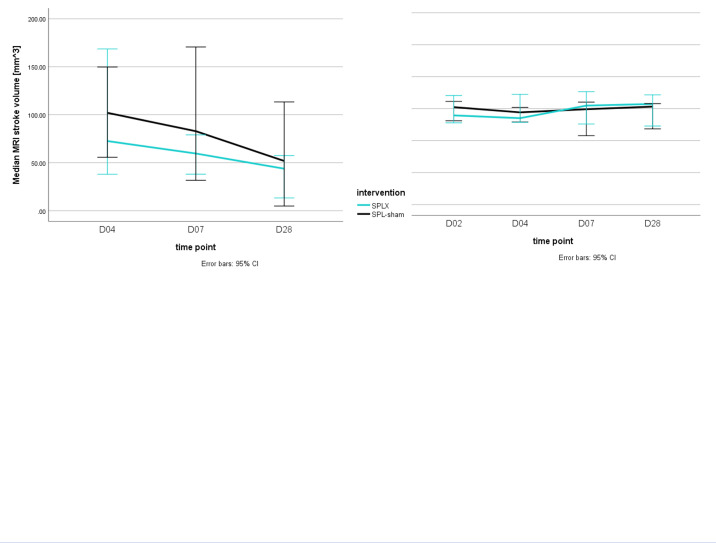
Dynamic changes in mice magnetic resonance imaging (MRI) brain characteristics on day two, four, seven, and 28 after middle cerebral artery occlusion (MCAO). Two groups of animals at multiple time points were compared using 95% confidence intervals. SPL-sham – sham operated mice for splenectomy; SPLX – splenectomized mice; D – day; MCAO – middle cerebral artery occlusion; SPL-sham – sham operated mice for splenectomy; SPLX – splenectomized mice; IL – ipsilateral hemisphere volume; CL – contralateral hemisphere volume.

MRI results revealed no significant difference in brain infarct size between the SPLX and SPLX-sham groups post-MCAO (F (2,24) = 0.267, P = 0.768). However, a significant difference in brain infarct size was observed in days following MCAO in both groups compared with baseline (F (2,24) = 48.42, P < 0.001). Consequently, there was no significant difference in ipsilateral and contralateral hemisphere volumes between the SPLX and SPLX-sham groups (*P* values of 0.499 and 0.450, respectively). However, a significant difference in ipsilateral and contralateral hemisphere volumes was noted in both groups over the 28-day period (*P* values of 0.001 and 0.031, respectively). These findings suggest that while splenectomy did not significantly affect brain volume after MCAO, stroke significantly affected it (Figure 5).

### Immunofluorescent histological analysis

There was a significant difference in GFAP expression in the ipsilateral brain hemisphere between the SPLX-sham (M = 190.8, SD = 689.953) and SPLX (M = 2322.517, SD = 500.388) groups (t(118) = -3.787, *P* = 0.001). However, in the contralateral hemisphere there was no significant difference in GFAP expression between the SPLX-sham (M = 189.133, SD = 712.314) and SPLX (M = 1959.183, SD = 784.575) groups (t(118) = -0.475, *P* = 0.635), which indicates that splenectomy had no effect on GFAP expression in the contralateral hemisphere compared to the sham-operated group.

There was a significant difference in anti-TLR2 antibodies in the ipsilateral hemisphere between the SPLX-sham (M = 2028.233, SD = 915.778) and SPLX (M = 2436.200, SD = 993.469) groups (t(118) = -2.339, *P* = 0.021). Similarly, significant differences in TLR2 expression were observed in the contralateral hemisphere between the SPLX-sham (M = 2100.3, SD = 869.069) and SPLX (M = 1419.467, SD = 534.943) groups (t(98.096) = 5.168, *P* < 0.001).

There was a significant difference in Iba-1 expression in the ipsilateral brain tissue between the SPLX-sham (M = 1786.650, SD = 592.384) and SPLX (M = 2329.017, SD = 806.350) groups (t(108.32) = -4.199, *P* < 0.001). The differences between the SPLX-sham (M = 1472.55, SD = 336.349) and SPLX (M = 1756.167, SD = 569.513) groups (t(102.855) = -3.321, *P* < 0.001) were also significant in the contralateral hemisphere.

Significant differences were also observed between the SPLX-sham and SPLX groups in CD68 expression in the ipsilateral (t(118) = -4.539, *P* < 0.001) and contralateral (t(102.855) = -3.758, *P* < 0.001) brain hemispheres, suggesting that splenectomy increased CD68 expression in both brain hemispheres.

**Figure 6:**
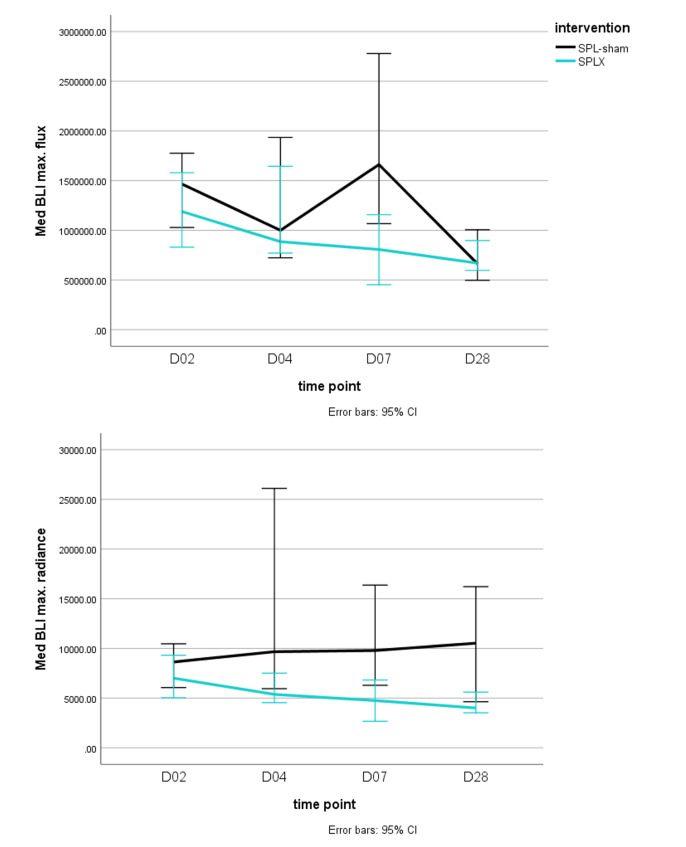
Imunohistological biomarkers in ipsi- (IL) and contralateral (CL) hemispheres of mice from both groups after middle cerebral artery occlusion (MCAO). We used *t* test for testing the statistical differences between the groups for every molecule that was assessed. Outliers are also shown in the plot, and each was designated for a better overview. A) Toll-like receptor 2 (TLR2); B) Glial fibrillary acidic protein (GFAP); C) Cluster of differentiation 68 (CD68); D) ionized calcium-binding adapter molecule 1 (Iba1); E) neuronal nuclear antigen (NeuN).

Significant differences in NeuN expression were observed between the SPLX-sham (M = 2145.133, SD = 575.263) and SPLX (M = 1736.5, SD = 588.884) groups (t(118) = 3.845, *P* < 0.001), indicating higher NeuN expression in the SPLX-sham group. Similarly, the analysis of the contralateral hemisphere revealed significant differences in NeuN expression between the SPLX-sham (M = 1958.95, SD = 713.367) and SPLX (M = 1533.783, SD = 527.117) groups (t(108.632) = 3.712, *P* < 0.001), indicating higher NeuN expression in the sham-operated group (Figure 6).

### Bioluminescence

**Figure 7:**
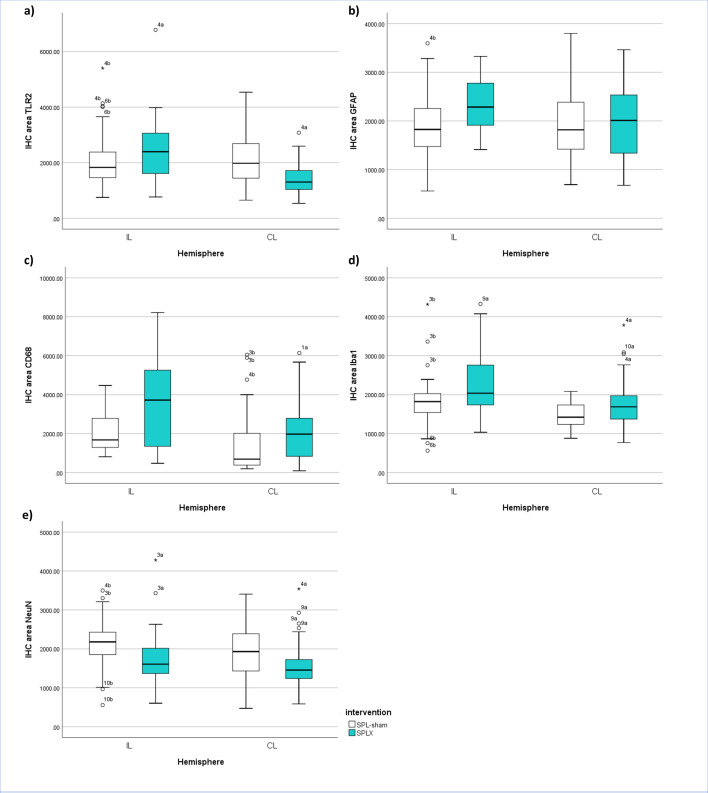
Dynamic changes in mice maximum radiance and maximum flux of bioluminescence (BLI) in both groups on day two, four, seven, and 28 after middle cerebral artery occlusion (MCAO). Two groups of animals were compared using 95% confidence intervals to compare two groups of animals at multiple time points. SPL-sham – sham operated mice for splenectomy; SPLX – splenectomized mice.

The analysis revealed a significant maximum increase in bioluminescence across time points (F(2,194, 13.165) = 5.933, *P* = 0.013), indicating a notable time effect. However, there was no significant effect when comparing the SPLX-sham group and the SPLX group (F(2,194, 13.165) = 0.825, *P* = 0.470). Regarding the maximum bioluminescent radiance, the analysis showed a significant effect across time points (F(2,331, 13.986) = 5.121, *P* = 0.018), but no significant difference between the SPLX-sham and SPLX groups (F(2,331, 13.986) = 0.958, *P* = 0.420). Overall, these results suggest significant differences in the maximum increase in bioluminescence and radiance across time points, but no significant differences between the time point and intervention (Figure 7).

### Accuracy of SPLX and SPL-sham class predictions using ANN based on excluding individual variables and their combinations

In examining the accuracy of ANN predictions for SPLX and SPL-sham classes, we assessed the impact of excluding individual variables and their combinations. Analyzing data sets encompassing various post-MCAO days, we observed fluctuations in predictive outcomes based on excluded variables. Notably, the inclusion of all variables yielded the highest accuracy, while exclusions, such as those of MR volume measurements and peak BLI radiation, influenced the predictive performance. Subsequent analyses extending to different post-MCAO day combinations revealed subtle relationships between excluded variables, their combinations, and the resulting accuracy in predicting SPLX and SPL-sham classes. The statistical significance of these differences highlights the complexity of ANN performance under varying conditions, enhancing our understanding of the model's sensitivity to input variations.

### ANN learning outcomes on the data set encompassing all post-stroke days (second, fourth, seventh, and 28th day)

The accuracy of predicting SPLX mice group membership was highest when the model was trained on a data set containing all variables (0.7736 ± 0.0234) (Supplemental Table 1(Supplementary Table 1)). The lowest accuracy in predicting the SPLX class was observed when the model was trained on a data set excluding the combination of variables MR IL/MR CL/animal mass/NS/BL peak increment/BL peak radiation (0.4134 ± 0.1784) (Supplemental Table 1(Supplementary Table 1)).

The highest accuracy in predicting the SPL-sham class was achieved when the model was trained on a data set excluding the variable combination MR IL/MR CL/animal mass (0.8321 ± 0.0277) (Supplemental Table 2(Supplementary Table 2)). The lowest accuracy in predicting the SPL-sham class was observed when the model was trained on a data set excluding the variable combination MR IL/MR CL/animal mass/NS/BL peak increment/BL peak radiation (0.3136 ± 0.2362) (Supplemental Table 2(Supplementary Table 2)).

The likelihood of a significant difference, that is, the accuracy of predicting the affiliation of splenectomized mice by ANN trained on data sets excluding variables or their combinations compared with ANN trained using the complete data set was determined for approximately 81% of variable combinations (102/126). A statistically significant difference in predicting the SPL-sham class accuracy by ANN trained on data sets excluding variables or their combinations compared with ANN trained using the complete data set was found for approximately 63% of variable combinations (80/126).

A significant difference in the accuracy of predicting SPLX and SPL-sham classes was noted in approximately 86% (109/127) of cases by excluding variables and their combinations. Here, the accuracy of predicting the SPLX class was significantly higher compared with predicting the SPL-sham class in 20 cases, while in the remaining 89 cases, the accuracy of predicting the SPL-sham class was significantly higher compared with the accuracy of predicting the SPLX class (Supplemental Table 3(Supplementary Table 3)).

The largest difference between the accuracy of predicting SPLX and SPL-sham classes (SPLX – SPL-sham) was observed when the model was trained on data sets excluding the variable combination day/MR IL/MR CL/animal mass/BL peak increment/BL peak radiation, amounting to 0.1742 (Supplemental Table 3(Supplementary Table 3)). The most substantial difference between predicting SPL-sham and SPLX classes (SPL-sham – SPLX) was found when the model was trained on data sets excluding the variable combination day/MR IL/animal mass/NS/BL peak increment/BL peak radiation, amounting to 0.1237 (Supplemental Table 3(Supplementary Table 3)).

### ANN learning results from the data set with all days post-stroke except day 2 (fourth, seventh, and 28th day)

The accuracy in predicting the SPLX class was highest when the model was trained on a data set containing all variables (0.8485 ± 0.0254) (Supplemental Table 4(Supplementary Table 4)). The lowest accuracy for predicting the SPLX class was observed after training on a data set excluding the variable combination MR IL/MR CL/animal mass/NS/BL peak increment/BL peak radiation (0.3081 ± 0.232) (Supplemental Table 4(Supplementary Table 4)).

The accuracy in predicting the SPL-sham class was highest when the model was trained on a data set excluding the variable combination MR CL/animal mass/NS (0.9284 ± 0.0366) (Supplemental Table 5(Supplementary Table 5)). The lowest accuracy for predicting the SPL-sham class was observed after training on a data set excluding the variable combination MR IL/MR CL/animal mass/NS/BL peak increment/BL peak radiation (0.4299 ± 0.1477) (Supplemental Table 5(Supplementary Table 5)).

A significant difference in the accuracy of predicting the SPLX class by ANN trained on data sets excluding variables or their combinations compared with ANN trained on the complete data set was identified for about 87% of variable combinations (110/126). Similarly, a significant difference in the accuracy of predicting the SPL-sham class was identified for approximately 66% of variable combinations (83/126).

Significant differences in predicting the SPLX and SPL-sham classes were noted in roughly 82% (104/127) of cases by excluding variables and their combinations. In these cases, the accuracy in predicting the SPLX class was significantly higher in 13 cases, while in the remaining 91 cases, the accuracy in predicting the SPL-sham class was significantly higher than predicting the SPLX class (Supplemental Table 6(Supplementary Table 6))

The largest difference between predicting the SPLX and SPL-sham classes (SPLX – SPL-sham) was observed when the model was trained on a data set excluding the variable combination day/MR IL/MR CL/animal mass/NS/BL peak radiation, amounting to 0.2269. The largest difference between predicting the SPL-sham and SPLX classes (SPL-sham – SPLX) was found when the model was trained on a data set excluding the variable combination day/MR IL/animal mass/NS/BL peak increment/BL peak radiation, amounting to 0.1541 (Supplemental Table 6(Supplementary Table 6)).

### ANN learning results from the data set with all days post-stroke except day 2 and day 4 (seventh and 28th days)

The accuracy in predicting the SPLX class was highest when the model was trained on a data set excluding the variable combination MR IL/MR CL/animal mass/NS' (0.9156 ± 0.0303) (Supplemental Table 7(Supplementary Table 7)). The lowest accuracy in predicting the SPLX class was observed after training on a data set excluding the variable combination MR IL/MR CL/animal mass/NS/BL peak increment/BL peak radiation (0.3792 ± 0.1951) (Supplemental Table 7(Supplementary Table 7)).

The accuracy in predicting the SPLX-sham class was highest when trained on a data set excluding the variable combination MR IL/animal mass (0.9867 ± 0.0154). The lowest accuracy in predicting the SPLX-sham class was observed after training on a data set excluding the variable combination MR IL/MR CL/animal mass/NS/BL peak increment/BL peak radiation (0.4190 ± 0.1456) (Supplemental Table 8(Supplementary Table 8)).

A significant difference in the accuracy of predicting the SPLX class by ANN trained on data sets excluding variables or their combinations compared with ANN trained on the complete data set was found for about 57% of variable combinations (72/126). Similarly, a significant difference in the accuracy of predicting the SPL-sham class was found for approximately 50% of variable combinations (63/126).

Significant differences in predicting the SPLX and SPL-sham classes were noted in roughly 83% (106/127) of cases by excluding variables and their combinations. In these cases, the accuracy in predicting the SPLX class was significantly higher in 12 cases, while in the remaining 94 cases, the accuracy in predicting the SPL-sham class was significantly higher than predicting the SPLX class (Supplemental Table 9(Supplementary Table 9)).

The largest difference between predicting the SPLX and SPL-sham classes (SPLX - SPL-sham) was observed when the model was trained on a data set excluding the variable combination day/MR IL/MR CL/animal mass/NS/BL peak radiation, amounting to 0.2346. The largest difference between predicting the SPL-sham and SPLX classes (SPL-sham - SPLX) was found when the model was trained on a data set excluding the variable combination day/MR IL/animal mass/NS/BL peak increment/BL peak radiation, amounting to 0.1314 (Supplemental Table 9(Supplementary Table 9)).

## Discussion

This study successfully confirmed the initial hypothesis that without increasing the number of subjects in the experiment, training neural networks demonstrated the separation of groups although most differences were not evident through frequentist statistics. We also confirmed that splenectomy, likely due to the reduction in the number of monocytes in the area of the MCAO-induced ischemic lesion, measurably reduced lesion size and consequently acted neuroprotectively. This aligns with previous studies on similar animal models (40). Consequently, the novelty of this research is the ability to yield such results with a relatively small number of animals and by adhering to stricter ethical principles in animal research but using a slightly different statistical model (in this case the ANN).

This research contributes to the knowledge of ischemic stroke treatment as cerebrovascular incidents remain the second leading global cause of mortality and a major cause of permanent disability. Currently, the only approved treatments for acute ischemic stroke involve restoring cerebral blood flow, including intravenous alteplase administration, and mechanical thrombectomy. These therapies come with significant limitations, such as narrow therapeutic time frames, strict inclusion criteria, and the potential for life-threatening complications. As a result, the quest for new medical interventions with neuroprotective and/or neurorestorative properties is an important future goal in cerebrovascular disease research, and one potential intervention could involve the functional exclusion of the spleen and/or its mediators from the equation (41).

Among various pathogenetic mechanisms in cerebral ischemic changes, inflammation emerges as a potential therapeutic target, given its involvement in nearly all aspects of post-ischemic damage and recovery. After a stroke, microglia quickly activate alongside various other types of cells that infiltrate the brain through peripheral blood vessels. Circulating monocytes/macrophages constitute one such cell lineage, whose presence in the post-ischemic brain peaks 3-7 days after inducing a stroke in mice. These murine monocytes are known to participate in multiple inflammatory processes characterized by two opposing phenotypes: the pro-inflammatory Ly6Chigh phenotype recruited via C-C chemokine receptor 2 and the anti-inflammatory Ly6Clow phenotype showing high expression of CX3C chemokine receptor 1. However, their exact roles in stroke-induced damage and subsequent recovery are yet to be clarified (41).

When analyzing the results of neurological behavioral tests, we used a standard scoring system to assess neurological outcomes in mice after inducing a stroke. Although there was no significant difference in neurological outcomes immediately after stroke induction, we noticed a significant difference in the results 28 days later. This clearly indicates the impact of MCAO induction on the neurological outcomes of mice, while on the other hand, splenectomy or sham surgery did not have a significant effect on their overall neurological function. However, by observing the curves, we noticed some separation between the groups based on days, but the frequentist analysis did not yield significant results. A similar effect was observed when comparing hemisphere volumes and ischemia area measured by MRI volumetry; although the groups seemed statistically different, after conducting actual frequentist statistical tests, no difference was found between the groups.

Furthermore, the mean area of histological immunofluorescence for the sham-operated group was smaller compared with the splenectomy group. Additionally, a significant difference in TLR2 expression was found in the ipsilateral brain hemisphere after splenectomy. Moreover, the analysis of immunofluorescence in the ipsilateral hemispheres revealed significant differences between the sham-operated group and the splenectomy group in terms of Iba-1 markers and CD68 expression. Additionally, stronger NeuN expression was observed in the sham-operated group compared with the splenectomy group, indicating the potential role of the spleen in preserving neurons after a stroke. All this strongly suggests a possible influence of the spleen on microglial activation and changes in cellular activity after a stroke, consistent with previous research confirming this premise.

The splenectomy group had significantly lower bioluminescence at two, seven, and 14 days compared with the sham group. This demonstrates the effect of splenectomy on the inflammatory response after an ischemic stroke, supporting the notion that splenectomy reduces inflammation, which might contribute to the observed reduction in lesion size and neuroprotection.

This study has several limitations. The first may result from the homogeneity of the mouse sample (eg, all mice were of the same age, genotype or from the same breeding environment). Differences in genetic material, age and environmental conditions can significantly affect the results and limit the transferability of the results to other populations. Furthermore, neural networks can be “black boxes,” ie, it is sometimes difficult to interpret how and why the model makes certain predictions. This can be a limitation, especially in a biomedical context where understanding the mechanisms behind the predictions and previous experience with similar experiments can be crucial. Additionally, if the model had been trained on a specific data set, its ability to generalize to other data sets may be limited. This is particularly important when there are significant differences between data sets, such as differences in splenectomy procedures, differences in treatments before and after surgery, or differences in measurements. Finally, depending on the performance of the model, limitations in accuracy (eg, high rates of false positives or false negatives) may affect the practical application of the model in real research or clinical settings.

In conclusion, our findings suggest that splenectomy could have a neuroprotective effect in acute ischemic stroke, potentially mediated through the modulation of inflammation and immune cell responses. But even more, through the sophisticated application of ANN, this study demonstrated the neuroprotective benefits of splenectomy in an MCAO model, even with a smaller number of animals. This result not only reinforces our understanding of splenectomy's therapeutic potential but also demonstrates the power of advanced statistical methodologies to optimize experimental designs in biomedical research, paving the way for more efficient and ethically more acceptable scientific studies.
