# Supplementary material for: Innovative statistical approaches: the use of neural networks reduces the sample size in the splenectomy-MCAO mouse model
Source: Croat Med J. 2024 Apr;65(2):122–37. doi: 10.3325/cmj.2024.65.122 (PMC11074938; doi:10.3325/cmj.2024.65.122)
Supplement: Supplementary Table 1 [file CroatMedJ_65_s001.pdf]

Supplemental Table 1. The prediction accuracy of the ANN SPLX class depending on the exclusion of variables and their combinations. The ANN was trained using a dataset with all days after a stroke. Values in the table are sorted based on the mean accuracy of SPLX class predictions.

"Out" variable	"In" variable	The mean accuracy value of ANN predictions for the SPLX class.	SD of accuracy in predictions for the ANN SPLX class.
None	Day_nr-MRI_IPSI-MRI_CONTRA-WEIGHT-NS-BLI_max_flux-BLI_max_radiance	0.7736	0.0234
MRI_CONTRA	Day_nr-MRI_IPSI-WEIGHT-NS-BLI_max_flux-BLI_max_radiance	0.7700	0.0257
MRI_IPSI	Day_nr-MRI_CONTRA-WEIGHT-NS-BLI_max_flux-BLI_max_radiance	0.7698	0.0258
BLI_max_radiance	Day_nr-MRI_IPSI-MRI_CONTRA-WEIGHT-NS-BLI_max_flux	0.7597	0.0223
WEIGHT	Day_nr-MRI_IPSI-MRI_CONTRA-NS-BLI_max_flux-BLI_max_radiance	0.7577	0.0236
Day_nr-MRI_IPSI-NS-BLI_max_flux	MRI_CONTRA-WEIGHT-BLI_max_radiance	0.7567	0.0440
MRI_IPSI-MRI_CONTRA	Day_nr-WEIGHT-NS-BLI_max_flux-BLI_max_radiance	0.7566	0.0256
MRI_CONTRA-BLI_max_radiance	Day_nr-MRI_IPSI-WEIGHT-NS-BLI_max_flux	0.7552	0.0239
MRI_IPSI-WEIGHT	Day_nr-MRI_CONTRA-NS-BLI_max_flux-BLI_max_radiance	0.7549	0.0238
MRI_IPSI-BLI_max_radiance	Day_nr-MRI_CONTRA-WEIGHT-NS-BLI_max_flux	0.7542	0.0240
NS	Day_nr-MRI_IPSI-MRI_CONTRA-WEIGHT-BLI_max_flux-BLI_max_radiance	0.7534	0.0350

Day_nr-MRI_IPSI-BLI_max_flux	MRI_CONTRA-WEIGHT-NS-BLI_max_radiance	0.7534	0.0405
MRI_CONTRA-WEIGHT	Day_nr-MRI_IPSI-NS-BLI_max_flux-BLI_max_radiance	0.7508	0.0241
Day_nr	MRI_IPSI-MRI_CONTRA-WEIGHT-NS-BLI_max_flux-BLI_max_radiance	0.7501	0.0297
Day_nr-BLI_max_radiance	MRI_IPSI-MRI_CONTRA-WEIGHT-NS-BLI_max_flux	0.7498	0.0311
Day_nr-MRI_IPSI-BLI_max_flux-BLI_max_radiance	MRI_CONTRA-WEIGHT-NS	0.7485	0.0292
MRI_CONTRA-NS	Day_nr-MRI_IPSI-WEIGHT-BLI_max_flux-BLI_max_radiance	0.7450	0.0304
NS-BLI_max_radiance	Day_nr-MRI_IPSI-MRI_CONTRA-WEIGHT-BLI_max_flux	0.7445	0.0344
MRI_IPSI-NS	Day_nr-MRI_CONTRA-WEIGHT-BLI_max_flux-BLI_max_radiance	0.7420	0.0340
WEIGHT-BLI_max_radiance	Day_nr-MRI_IPSI-MRI_CONTRA-NS-BLI_max_flux	0.7409	0.0247
MRI_IPSI-MRI_CONTRA-BLI_max_radiance	Day_nr-WEIGHT-NS-BLI_max_flux	0.7404	0.0221
BLI_max_flux	Day_nr-MRI_IPSI-MRI_CONTRA-WEIGHT-NS-BLI_max_radiance	0.7391	0.0308
MRI_IPSI-MRI_CONTRA-WEIGHT	Day_nr-NS-BLI_max_flux-BLI_max_radiance	0.7379	0.0258
Day_nr-NS-BLI_max_flux-BLI_max_radiance	MRI_IPSI-MRI_CONTRA-WEIGHT	0.7351	0.0335
Day_nr-MRI_IPSI-NS-BLI_max_flux-BLI_max_radiance	MRI_CONTRA-WEIGHT	0.7347	0.0326
MRI_IPSI-WEIGHT-BLI_max_radiance	Day_nr-MRI_CONTRA-NS-BLI_max_flux	0.7346	0.0220
Day_nr-NS	MRI_IPSI-MRI_CONTRA-WEIGHT-BLI_max_flux-BLI_max_radiance	0.7336	0.0310

MRI_IPSI-NS-BLI_max_flux-BLI_max_radiance	Day_nr-MRI_CONTRA-WEIGHT	0.7328	0.0314
WEIGHT-NS	Day_nr-MRI_IPSI-MRI_CONTRA-BLI_max_flux-BLI_max_radiance	0.7315	0.0329
Day_nr-BLI_max_flux-BLI_max_radiance	MRI_IPSI-MRI_CONTRA-WEIGHT-NS	0.7315	0.0262
Day_nr-MRI_CONTRA	MRI_IPSI-WEIGHT-NS-BLI_max_flux-BLI_max_radiance	0.7301	0.0287
MRI_CONTRA-NS-BLI_max_radiance	Day_nr-MRI_IPSI-WEIGHT-BLI_max_flux	0.7294	0.0324
MRI_CONTRA-BLI_max_flux	Day_nr-MRI_IPSI-WEIGHT-NS-BLI_max_radiance	0.7284	0.0349
Day_nr-NS-BLI_max_radiance	MRI_IPSI-MRI_CONTRA-WEIGHT-BLI_max_flux	0.7277	0.0422
Day_nr-MRI_IPSI-NS	MRI_CONTRA-WEIGHT-BLI_max_flux-BLI_max_radiance	0.7265	0.0568
Day_nr-WEIGHT	MRI_IPSI-MRI_CONTRA-NS-BLI_max_flux-BLI_max_radiance	0.7262	0.0287
BLI_max_flux-BLI_max_radiance	Day_nr-MRI_IPSI-MRI_CONTRA-WEIGHT-NS	0.7251	0.0268
Day_nr-NS-BLI_max_flux	MRI_IPSI-MRI_CONTRA-WEIGHT-BLI_max_radiance	0.7251	0.0532
Day_nr-BLI_max_flux	MRI_IPSI-MRI_CONTRA-WEIGHT-NS-BLI_max_radiance	0.7237	0.0332
Day_nr-MRI_CONTRA-BLI_max_radiance	MRI_IPSI-WEIGHT-NS-BLI_max_flux	0.7232	0.0265
MRI_CONTRA-WEIGHT-NS	Day_nr-MRI_IPSI-BLI_max_flux-BLI_max_radiance	0.7227	0.0365
MRI_IPSI-MRI_CONTRA-NS	Day_nr-WEIGHT-BLI_max_flux-BLI_max_radiance	0.7215	0.0258
Day_nr-MRI_IPSI-BLI_max_radiance	MRI_CONTRA-WEIGHT-NS-BLI_max_flux	0.7209	0.0633
MRI_IPSI-WEIGHT-NS	Day_nr-MRI_CONTRA-BLI_max_flux-BLI_max_radiance	0.7205	0.0330
Day_nr-MRI_IPSI-	WEIGHT-BLI_max_radiance	0.7199	0.0434

MRI_CONTRA-NS-BLI_max_flux			
MRI_IPSI-NS-BLI_max_radiance	Day_nr-MRI_CONTRA-WEIGHT-BLI_max_flux	0.7198	0.0349
Day_nr-MRI_IPSI-NS-BLI_max_radiance	MRI_CONTRA-WEIGHT-BLI_max_flux	0.7193	0.0519
Day_nr-MRI_IPSI	MRI_CONTRA-WEIGHT-NS-BLI_max_flux-BLI_max_radiance	0.7183	0.0543
MRI_IPSI-NS-BLI_max_flux	Day_nr-MRI_CONTRA-WEIGHT-BLI_max_radiance	0.7174	0.0552
Day_nr-WEIGHT-BLI_max_radiance	MRI_IPSI-MRI_CONTRA-NS-BLI_max_flux	0.7173	0.0298
Day_nr-MRI_CONTRA-NS	MRI_IPSI-WEIGHT-BLI_max_flux-BLI_max_radiance	0.7139	0.0330
MRI_IPSI-BLI_max_flux	Day_nr-MRI_CONTRA-WEIGHT-NS-BLI_max_radiance	0.7135	0.0293
WEIGHT-BLI_max_flux	Day_nr-MRI_IPSI-MRI_CONTRA-NS-BLI_max_radiance	0.7116	0.0299
MRI_IPSI-MRI_CONTRA-WEIGHT-NS	Day_nr-BLI_max_flux-BLI_max_radiance	0.7106	0.0257
NS-BLI_max_flux-BLI_max_radiance	Day_nr-MRI_IPSI-MRI_CONTRA-WEIGHT	0.7103	0.0267
MRI_CONTRA-WEIGHT-BLI_max_radiance	Day_nr-MRI_IPSI-NS-BLI_max_flux	0.7080	0.0180
Day_nr-MRI_CONTRA-NS-BLI_max_radiance	MRI_IPSI-WEIGHT-BLI_max_flux	0.7075	0.0325
MRI_IPSI-MRI_CONTRA-NS-BLI_max_radiance	Day_nr-WEIGHT-BLI_max_flux	0.7064	0.0241
MRI_IPSI-BLI_max_flux-BLI_max_radiance	Day_nr-MRI_CONTRA-WEIGHT-NS	0.7063	0.0281
Day_nr-WEIGHT-NS	MRI_IPSI-MRI_CONTRA-BLI_max_flux-BLI_max_radiance	0.7060	0.0352
Day_nr-MRI_CONTRA-BLI_max_flux	MRI_IPSI-WEIGHT-NS-BLI_max_radiance	0.7054	0.0270
Day_nr-MRI_IPSI-WEIGHT-	MRI_CONTRA-BLI_max_radiance	0.7052	0.0470

NS-BLI_max_flux			
WEIGHT-NS-BLI_max_radiance	Day_nr-MRI_IPSI-MRI_CONTRA-BLI_max_flux	0.7042	0.0407
MRI_CONTRA-WEIGHT-BLI_max_flux	Day_nr-MRI_IPSI-NS-BLI_max_radiance	0.7040	0.0262
Day_nr-MRI_CONTRA-WEIGHT	MRI_IPSI-NS-BLI_max_flux-BLI_max_radiance	0.7034	0.0298
MRI_IPSI-MRI_CONTRA-WEIGHT-BLI_max_radiance	Day_nr-NS-BLI_max_flux	0.7023	0.0265
MRI_IPSI-MRI_CONTRA-BLI_max_flux	Day_nr-WEIGHT-NS-BLI_max_radiance	0.7012	0.0282
Day_nr-MRI_IPSI-WEIGHT-NS-BLI_max_flux-BLI_max_radiance	MRI_CONTRA	0.7003	0.0341
NS-BLI_max_flux	Day_nr-MRI_IPSI-MRI_CONTRA-WEIGHT-BLI_max_radiance	0.6997	0.0307
MRI_IPSI-WEIGHT-NS-BLI_max_radiance	Day_nr-MRI_CONTRA-BLI_max_flux	0.6983	0.0331
MRI_CONTRA-BLI_max_flux-BLI_max_radiance	Day_nr-MRI_IPSI-WEIGHT-NS	0.6970	0.0229
Day_nr-WEIGHT-NS-BLI_max_radiance	MRI_IPSI-MRI_CONTRA-BLI_max_flux	0.6965	0.0363
Day_nr-WEIGHT-NS-BLI_max_flux-BLI_max_radiance	MRI_IPSI-MRI_CONTRA	0.6948	0.0464
WEIGHT-BLI_max_flux-BLI_max_radiance	Day_nr-MRI_IPSI-MRI_CONTRA-NS	0.6947	0.0230
MRI_IPSI-WEIGHT-BLI_max_flux	Day_nr-MRI_CONTRA-NS-BLI_max_radiance	0.6946	0.0260
Day_nr-MRI_CONTRA-WEIGHT-BLI_max_radiance	MRI_IPSI-NS-BLI_max_flux	0.6942	0.0287
MRI_IPSI-WEIGHT-NS-BLI_max_flux-BLI_max_radiance	Day_nr-MRI_CONTRA	0.6910	0.0343
Day_nr-MRI_CONTRA-NS-	MRI_IPSI-WEIGHT	0.6866	0.0394

BLI_max_flux-BLI_max_radiance			
Day_nr-WEIGHT-BLI_max_flux	MRI_IPSI-MRI_CONTRA-NS-BLI_max_radiance	0.6858	0.0310
Day_nr-WEIGHT-BLI_max_flux-BLI_max_radiance	MRI_IPSI-MRI_CONTRA-NS	0.6844	0.0280
Day_nr-MRI_CONTRA-WEIGHT-NS	MRI_IPSI-BLI_max_flux-BLI_max_radiance	0.6838	0.0342
Day_nr-MRI_CONTRA-BLI_max_flux-BLI_max_radiance	MRI_IPSI-WEIGHT-NS	0.6836	0.0239
Day_nr-MRI_CONTRA-NS-BLI_max_flux	MRI_IPSI-WEIGHT-BLI_max_radiance	0.6806	0.0371
MRI_IPSI-MRI_CONTRA-WEIGHT-BLI_max_flux	Day_nr-NS-BLI_max_radiance	0.6804	0.0301
MRI_CONTRA-WEIGHT-NS-BLI_max_radiance	Day_nr-MRI_IPSI-BLI_max_flux	0.6770	0.0355
MRI_CONTRA-NS-BLI_max_flux	Day_nr-MRI_IPSI-WEIGHT-BLI_max_radiance	0.6751	0.0209
MRI_CONTRA-NS-BLI_max_flux-BLI_max_radiance	Day_nr-MRI_IPSI-WEIGHT	0.6719	0.0273
Day_nr-MRI_CONTRA-WEIGHT-NS-BLI_max_radiance	MRI_IPSI-BLI_max_flux	0.6716	0.0343
Day_nr-MRI_IPSI-MRI_CONTRA-BLI_max_flux-BLI_max_radiance	WEIGHT-NS	0.6700	0.0673
WEIGHT-NS-BLI_max_flux	Day_nr-MRI_IPSI-MRI_CONTRA-BLI_max_radiance	0.6691	0.0475
MRI_IPSI-WEIGHT-BLI_max_flux-BLI_max_radiance	Day_nr-MRI_CONTRA-NS	0.6687	0.0268
Day_nr-MRI_CONTRA-WEIGHT-BLI_max_flux	MRI_IPSI-NS-BLI_max_radiance	0.6675	0.0303

Day_nr-MRI_IPSI-MRI_CONTRA-NS	WEIGHT-BLI_max_flux-BLI_max_radiance	0.6674	0.0312
WEIGHT-NS-BLI_max_flux-BLI_max_radiance	Day_nr-MRI_IPSI-MRI_CONTRA	0.6666	0.0418
Day_nr-MRI_IPSI-WEIGHT	MRI_CONTRA-NS-BLI_max_flux-BLI_max_radiance	0.6662	0.0287
Day_nr-MRI_IPSI-WEIGHT-BLI_max_radiance	MRI_CONTRA-NS-BLI_max_flux	0.6641	0.0259
MRI_IPSI-MRI_CONTRA-NS-BLI_max_flux-BLI_max_radiance	Day_nr-WEIGHT	0.6639	0.0290
MRI_IPSI-MRI_CONTRA-WEIGHT-NS-BLI_max_radiance	Day_nr-BLI_max_flux	0.6629	0.0214
MRI_IPSI-MRI_CONTRA-BLI_max_flux-BLI_max_radiance	Day_nr-WEIGHT-NS	0.6628	0.0265
MRI_CONTRA-WEIGHT-NS-BLI_max_flux	Day_nr-MRI_IPSI-BLI_max_radiance	0.6624	0.0427
MRI_IPSI-MRI_CONTRA-NS-BLI_max_flux	Day_nr-WEIGHT-BLI_max_radiance	0.6620	0.0463
Day_nr-MRI_IPSI-MRI_CONTRA	WEIGHT-NS-BLI_max_flux-BLI_max_radiance	0.6619	0.0244
Day_nr-MRI_IPSI-MRI_CONTRA-BLI_max_flux	WEIGHT-NS-BLI_max_radiance	0.6615	0.0429
Day_nr-WEIGHT-NS-BLI_max_flux	MRI_IPSI-MRI_CONTRA-BLI_max_radiance	0.6615	0.0414
Day_nr-MRI_IPSI-MRI_CONTRA-NS-BLI_max_flux-BLI_max_radiance	WEIGHT	0.6609	0.0232
Day_nr-MRI_IPSI-MRI_CONTRA-BLI_max_radiance	WEIGHT-NS-BLI_max_flux	0.6600	0.0231
Day_nr-MRI_IPSI-WEIGHT-NS	MRI_CONTRA-BLI_max_flux-BLI_max_radiance	0.6575	0.0350

MRI_IPSI-WEIGHT-NS-BLI_max_flux	Day_nr-MRI_CONTRA-BLI_max_radiance	0.6547	0.0423
Day_nr-MRI_IPSI-MRI_CONTRA-WEIGHT-BLI_max_radiance	NS-BLI_max_flux	0.6520	0.0242
Day_nr-MRI_IPSI-MRI_CONTRA-NS-BLI_max_radiance	WEIGHT-BLI_max_flux	0.6519	0.0341
Day_nr-MRI_IPSI-WEIGHT-BLI_max_flux	MRI_CONTRA-NS-BLI_max_radiance	0.6509	0.0458
MRI_CONTRA-WEIGHT-BLI_max_flux-BLI_max_radiance	Day_nr-MRI_IPSI-NS	0.6496	0.0279
Day_nr-MRI_IPSI-WEIGHT-NS-BLI_max_radiance	MRI_CONTRA-BLI_max_flux	0.6484	0.0459
Day_nr-MRI_IPSI-MRI_CONTRA-WEIGHT	NS-BLI_max_flux-BLI_max_radiance	0.6476	0.0258
Day_nr-MRI_CONTRA-WEIGHT-BLI_max_flux-BLI_max_radiance	MRI_IPSI-NS	0.6417	0.0251
Day_nr-MRI_IPSI-MRI_CONTRA-WEIGHT-NS	BLI_max_flux-BLI_max_radiance	0.6400	0.0257
Day_nr-MRI_IPSI-WEIGHT-BLI_max_flux-BLI_max_radiance	MRI_CONTRA-NS	0.6287	0.0407
MRI_IPSI-MRI_CONTRA-WEIGHT-NS-BLI_max_flux	Day_nr-BLI_max_radiance	0.6287	0.0388
Day_nr-MRI_IPSI-MRI_CONTRA-WEIGHT-BLI_max_flux	NS-BLI_max_radiance	0.6145	0.0278
MRI_IPSI-MRI_CONTRA-WEIGHT-BLI_max_flux-BLI_max_radiance	Day_nr-NS	0.6141	0.0125
Day_nr-MRI_CONTRA-WEIGHT-NS-BLI_max_flux	MRI_IPSI-BLI_max_radiance	0.6073	0.0312
Day_nr-MRI_IPSI-MRI_CONTRA-WEIGHT-NS-	BLI_max_flux	0.6036	0.0184

BLI_max_radiance			
Day_nr-MRI_IPSI-MRI_CONTRA-WEIGHT-BLI_max_flux-BLI_max_radiance	NS	0.5859	0.0129
MRI_CONTRA-WEIGHT-NS-BLI_max_flux-BLI_max_radiance	Day_nr-MRI_IPSI	0.5714	0.0264
Day_nr-MRI_IPSI-MRI_CONTRA-WEIGHT-NS-BLI_max_flux	BLI_max_radiance	0.5619	0.0235
Day_nr-MRI_CONTRA-WEIGHT-NS-BLI_max_flux-BLI_max_radiance	MRI_IPSI	0.5587	0.0258
MRI_IPSI-MRI_CONTRA-WEIGHT-NS-BLI_max_flux-BLI_max_radiance	Day_nr	0.4134	0.1784

ANN - artificial neural network; SPLX - splenectomized mice group; SD - standard deviation; MRI_CONTRA - volume of the contralateral hemisphere measured by MRI; MRI_IPSI - volume of the ipsilateral hemisphere measured by MRI; BLI_max_radiance - area of peak radiation measured by bioluminescence method; BLI_max_flux - area of peak growth measured by bioluminescence method; WEIGHT - animal weight; Day_nr - day from the middle cerebral artery occlusion (MCAO) procedure; NS - scoring of phenotypic neurological assessment.
