# Supplementary material for: Innovative statistical approaches: the use of neural networks reduces the sample size in the splenectomy-MCAO mouse model
Source: Croat Med J. 2024 Apr;65(2):122–37. doi: 10.3325/cmj.2024.65.122 (PMC11074938; doi:10.3325/cmj.2024.65.122)
Supplement: Supplementary Table 2 [file CroatMedJ_65_s002.pdf]

Supplemental Table 2. The prediction accuracy of the ANN SPL-sham class depending on the exclusion of variables and their combinations. The ANN was trained using a dataset with all days after a stroke. Values in the table are sorted based on the mean accuracy of SPL-sham class predictions.

"Out" variable	"In" variable	The mean accuracy value of ANN predictions for the SPL-sham class	SD of accuracy in predictions for the ANN SPL-sham class
MRI_IPSI-MRI_CONTRA-WEIGHT	Day_nr-NS-BLI_max_flux-BLI_max_radiance	0.8321	0.0277
MRI_CONTRA-WEIGHT	Day_nr-MRI_IPSI-NS-BLI_max_flux-BLI_max_radiance	0.8218	0.0272
MRI_IPSI-WEIGHT	Day_nr-MRI_CONTRA-NS-BLI_max_flux-BLI_max_radiance	0.8161	0.0390
MRI_IPSI-MRI_CONTRA-WEIGHT-NS	Day_nr-BLI_max_flux-BLI_max_radiance	0.8146	0.0328
MRI_IPSI-MRI_CONTRA	Day_nr-WEIGHT-NS-BLI_max_flux-BLI_max_radiance	0.8088	0.0299
MRI_CONTRA-WEIGHT-NS	Day_nr-MRI_IPSI-BLI_max_flux-BLI_max_radiance	0.8084	0.0450
MRI_IPSI	Day_nr-MRI_CONTRA-WEIGHT-NS-BLI_max_flux-BLI_max_radiance	0.8074	0.0265
MRI_IPSI-MRI_CONTRA-WEIGHT-BLI_max_flux	Day_nr-NS-BLI_max_radiance	0.8004	0.0372
None	Day_nr-MRI_IPSI-MRI_CONTRA-WEIGHT-NS-BLI_max_flux-BLI_max_radiance	0.8000	0.0264
WEIGHT	Day_nr-MRI_IPSI-	0.7997	0.0300

	MRI_CONTRA-NS-BLI_max_flux-BLI_max_radiance		
MRI_CONTRA	Day_nr-MRI_IPSI-WEIGHT-NS-BLI_max_flux-BLI_max_radiance	0.7985	0.0276
MRI_CONTRA-WEIGHT-BLI_max_flux	Day_nr-MRI_IPSI-NS-BLI_max_radiance	0.7975	0.0409
MRI_IPSI-WEIGHT-NS	Day_nr-MRI_CONTRA-BLI_max_flux-BLI_max_radiance	0.7944	0.0474
MRI_IPSI-BLI_max_radiance	Day_nr-MRI_CONTRA-WEIGHT-NS-BLI_max_flux	0.7875	0.0271
MRI_CONTRA-WEIGHT-BLI_max_radiance	Day_nr-MRI_IPSI-NS-BLI_max_flux	0.7875	0.0263
NS	Day_nr-MRI_IPSI-MRI_CONTRA-WEIGHT-BLI_max_flux-BLI_max_radiance	0.7859	0.0272
WEIGHT-NS	Day_nr-MRI_IPSI-MRI_CONTRA-BLI_max_flux-BLI_max_radiance	0.7859	0.0449
Day_nr-MRI_IPSI-MRI_CONTRA-WEIGHT-BLI_max_radiance	NS-BLI_max_flux	0.7852	0.0332
MRI_IPSI-MRI_CONTRA-WEIGHT-NS-BLI_max_flux	Day_nr-BLI_max_radiance	0.7852	0.0383
MRI_IPSI-NS	Day_nr-MRI_CONTRA-WEIGHT-BLI_max_flux-BLI_max_radiance	0.7849	0.0279
MRI_IPSI-MRI_CONTRA-BLI_max_radiance	Day_nr-WEIGHT-NS-BLI_max_flux	0.7837	0.0244
BLI_max_radiance	Day_nr-MRI_IPSI-MRI_CONTRA-WEIGHT-	0.7832	0.0313

	NS-BLI_max_flux		
Day_nr-MRI_IPSI-WEIGHT-BLI_max_radiance	MRI_CONTRA-NS-BLI_max_flux	0.7822	0.0322
MRI_CONTRA-WEIGHT-NS-BLI_max_flux	Day_nr-MRI_IPSI-BLI_max_radiance	0.7791	0.0551
MRI_CONTRA-BLI_max_radiance	Day_nr-MRI_IPSI-WEIGHT-NS-BLI_max_flux	0.7778	0.0297
Day_nr-MRI_IPSI-MRI_CONTRA-BLI_max_radiance	WEIGHT-NS-BLI_max_flux	0.7762	0.0337
MRI_IPSI-MRI_CONTRA-WEIGHT-BLI_max_radiance	Day_nr-NS-BLI_max_flux	0.7739	0.0332
Day_nr-BLI_max_radiance	MRI_IPSI-MRI_CONTRA-WEIGHT-NS-BLI_max_flux	0.7731	0.0285
MRI_IPSI-MRI_CONTRA-NS	Day_nr-WEIGHT-BLI_max_flux-BLI_max_radiance	0.7731	0.0256
MRI_IPSI-WEIGHT-BLI_max_radiance	Day_nr-MRI_CONTRA-NS-BLI_max_flux	0.7729	0.0333
Day_nr	MRI_IPSI-MRI_CONTRA-WEIGHT-NS-BLI_max_flux-BLI_max_radiance	0.7720	0.0399
MRI_CONTRA-NS	Day_nr-MRI_IPSI-WEIGHT-BLI_max_flux-BLI_max_radiance	0.7717	0.0322
WEIGHT-BLI_max_radiance	Day_nr-MRI_IPSI-MRI_CONTRA-NS-BLI_max_flux	0.7687	0.0340
Day_nr-MRI_CONTRA-BLI_max_radiance	MRI_IPSI-WEIGHT-NS-BLI_max_flux	0.7671	0.0383
BLI_max_flux	Day_nr-MRI_IPSI-MRI_CONTRA-WEIGHT-NS-BLI_max_radiance	0.7658	0.0388
MRI_CONTRA-	Day_nr-MRI_IPSI-WEIGHT-	0.7650	0.0360

BLI_max_flux	NS-BLI_max_radiance		
Day_nr-MRI_CONTRA	MRI_IPSI-WEIGHT-NS-BLI_max_flux-BLI_max_radiance	0.7649	0.0360
WEIGHT-BLI_max_flux	Day_nr-MRI_IPSI-MRI_CONTRA-NS-BLI_max_radiance	0.7644	0.0370
MRI_IPSI-MRI_CONTRA-BLI_max_flux	Day_nr-WEIGHT-NS-BLI_max_radiance	0.7644	0.0357
Day_nr-WEIGHT	MRI_IPSI-MRI_CONTRA-NS-BLI_max_flux-BLI_max_radiance	0.7631	0.0288
MRI_IPSI-BLI_max_flux	Day_nr-MRI_CONTRA-WEIGHT-NS-BLI_max_radiance	0.7618	0.0338
Day_nr-WEIGHT-BLI_max_radiance	MRI_IPSI-MRI_CONTRA-NS-BLI_max_flux	0.7612	0.0290
Day_nr-MRI_IPSI-MRI_CONTRA-WEIGHT-BLI_max_flux-BLI_max_radiance	NS	0.7601	0.0343
Day_nr-MRI_IPSI-WEIGHT	MRI_CONTRA-NS-BLI_max_flux-BLI_max_radiance	0.7597	0.0292
MRI_IPSI-MRI_CONTRA-WEIGHT-NS-BLI_max_radiance	Day_nr-BLI_max_flux	0.7593	0.0239
NS-BLI_max_radiance	Day_nr-MRI_IPSI-MRI_CONTRA-WEIGHT-BLI_max_flux	0.7586	0.0217
MRI_IPSI-WEIGHT-BLI_max_flux	Day_nr-MRI_CONTRA-NS-BLI_max_radiance	0.7570	0.0485
MRI_IPSI-NS-BLI_max_radiance	Day_nr-MRI_CONTRA-WEIGHT-BLI_max_flux	0.7558	0.0249

MRI_IPSI-WEIGHT-NS-BLI_max_flux	Day_nr-MRI_CONTRA-BLI_max_radiance	0.7552	0.0598
Day_nr-MRI_CONTRA-WEIGHT-BLI_max_radiance	MRI_IPSI-NS-BLI_max_flux	0.7511	0.0271
Day_nr-MRI_CONTRA-WEIGHT	MRI_IPSI-NS-BLI_max_flux-BLI_max_radiance	0.7503	0.0338
MRI_CONTRA-NS-BLI_max_radiance	Day_nr-MRI_IPSI-WEIGHT-BLI_max_flux	0.7497	0.0298
MRI_IPSI-MRI_CONTRA-NS-BLI_max_radiance	Day_nr-WEIGHT-BLI_max_flux	0.7493	0.0303
Day_nr-MRI_IPSI-MRI_CONTRA-WEIGHT	NS-BLI_max_flux-BLI_max_radiance	0.7457	0.0380
Day_nr-MRI_CONTRA-BLI_max_flux	MRI_IPSI-WEIGHT-NS-BLI_max_radiance	0.7456	0.0379
WEIGHT-NS-BLI_max_flux	Day_nr-MRI_IPSI-MRI_CONTRA-BLI_max_radiance	0.7434	0.0422
Day_nr-NS	MRI_IPSI-MRI_CONTRA-WEIGHT-BLI_max_flux-BLI_max_radiance	0.7413	0.0315
Day_nr-MRI_CONTRA-NS-BLI_max_radiance	MRI_IPSI-WEIGHT-BLI_max_flux	0.7408	0.0308
MRI_CONTRA-NS-BLI_max_flux	Day_nr-MRI_IPSI-WEIGHT-BLI_max_radiance	0.7397	0.0222
Day_nr-MRI_CONTRA-NS	MRI_IPSI-WEIGHT-BLI_max_flux-BLI_max_radiance	0.7394	0.0333
Day_nr-NS-BLI_max_radiance	MRI_IPSI-MRI_CONTRA-WEIGHT-BLI_max_flux	0.7360	0.0244
Day_nr-MRI_CONTRA-WEIGHT-BLI_max_flux	MRI_IPSI-NS-BLI_max_radiance	0.7360	0.0395
Day_nr-MRI_IPSI-MRI_CONTRA	WEIGHT-NS-BLI_max_flux-BLI_max_radiance	0.7341	0.0462

Day_nr-WEIGHT-NS	MRI_IPSI-MRI_CONTRA-BLI_max_flux-BLI_max_radiance	0.7333	0.0305
Day_nr-MRI_IPSI	MRI_CONTRA-WEIGHT-NS-BLI_max_flux-BLI_max_radiance	0.7325	0.0431
Day_nr-MRI_CONTRA-WEIGHT-NS	MRI_IPSI-BLI_max_flux-BLI_max_radiance	0.7306	0.0357
MRI_CONTRA-WEIGHT-NS-BLI_max_radiance	Day_nr-MRI_IPSI-BLI_max_flux	0.7296	0.0302
Day_nr-MRI_IPSI-MRI_CONTRA-WEIGHT-NS-BLI_max_radiance	BLI_max_flux	0.7283	0.0211
Day_nr-BLI_max_flux	MRI_IPSI-MRI_CONTRA-WEIGHT-NS-BLI_max_radiance	0.7275	0.0381
NS-BLI_max_flux	Day_nr-MRI_IPSI-MRI_CONTRA-WEIGHT-BLI_max_radiance	0.7266	0.0404
Day_nr-MRI_IPSI-WEIGHT-NS	MRI_CONTRA-BLI_max_flux-BLI_max_radiance	0.7266	0.0309
Day_nr-WEIGHT-NS-BLI_max_radiance	MRI_IPSI-MRI_CONTRA-BLI_max_flux	0.7265	0.0261
WEIGHT-NS-BLI_max_radiance	Day_nr-MRI_IPSI-MRI_CONTRA-BLI_max_flux	0.7259	0.0308
Day_nr-WEIGHT-BLI_max_flux	MRI_IPSI-MRI_CONTRA-NS-BLI_max_radiance	0.7258	0.0353
Day_nr-MRI_IPSI-MRI_CONTRA-WEIGHT-BLI_max_flux	NS-BLI_max_radiance	0.7234	0.0398
Day_nr-MRI_IPSI-MRI_CONTRA-NS	WEIGHT-BLI_max_flux-BLI_max_radiance	0.7232	0.0301

BLI_max_flux-BLI_max_radiance	Day_nr-MRI_IPSI-MRI_CONTRA-WEIGHT-NS	0.7219	0.0358
Day_nr-MRI_CONTRA-WEIGHT-NS-BLI_max_radiance	MRI_IPSI-BLI_max_flux	0.7208	0.0328
Day_nr-MRI_IPSI-MRI_CONTRA-NS-BLI_max_radiance	WEIGHT-BLI_max_flux	0.7205	0.0459
MRI_CONTRA-BLI_max_flux-BLI_max_radiance	Day_nr-MRI_IPSI-WEIGHT-NS	0.7203	0.0348
Day_nr-MRI_IPSI-MRI_CONTRA-WEIGHT-NS	BLI_max_flux-BLI_max_radiance	0.7200	0.0310
MRI_IPSI-WEIGHT-NS-BLI_max_radiance	Day_nr-MRI_CONTRA-BLI_max_flux	0.7199	0.0311
Day_nr-MRI_IPSI-WEIGHT-BLI_max_flux	MRI_CONTRA-NS-BLI_max_radiance	0.7184	0.0305
MRI_IPSI-MRI_CONTRA-WEIGHT-BLI_max_flux-BLI_max_radiance	Day_nr-NS	0.7168	0.0269
Day_nr-MRI_IPSI-MRI_CONTRA-BLI_max_flux	WEIGHT-NS-BLI_max_radiance	0.7161	0.0233
MRI_CONTRA-WEIGHT-BLI_max_flux-BLI_max_radiance	Day_nr-MRI_IPSI-NS	0.7159	0.0386
Day_nr-MRI_IPSI-MRI_CONTRA-WEIGHT-NS-BLI_max_flux	BLI_max_radiance	0.7158	0.0226
WEIGHT-BLI_max_flux-BLI_max_radiance	Day_nr-MRI_IPSI-MRI_CONTRA-NS	0.7146	0.0431
Day_nr-WEIGHT-BLI_max_flux-BLI_max_radiance	MRI_IPSI-MRI_CONTRA-NS	0.7125	0.0391

Day_nr-MRI_CONTRA-WEIGHT-BLI_max_flux-BLI_max_radiance	MRI_IPSI-NS	0.7104	0.0356
Day_nr-MRI_IPSI-WEIGHT-NS-BLI_max_radiance	MRI_CONTRA-BLI_max_flux	0.7096	0.0437
MRI_IPSI-WEIGHT-BLI_max_flux-BLI_max_radiance	Day_nr-MRI_CONTRA-NS	0.7085	0.0334
Day_nr-MRI_IPSI-BLI_max_radiance	MRI_CONTRA-WEIGHT-NS-BLI_max_flux	0.7073	0.0599
Day_nr-MRI_CONTRA-BLI_max_flux-BLI_max_radiance	MRI_IPSI-WEIGHT-NS	0.7070	0.0365
MRI_IPSI-BLI_max_flux-BLI_max_radiance	Day_nr-MRI_CONTRA-WEIGHT-NS	0.6996	0.0369
Day_nr-MRI_IPSI-NS	MRI_CONTRA-WEIGHT-BLI_max_flux-BLI_max_radiance	0.6972	0.0399
MRI_IPSI-MRI_CONTRA-NS-BLI_max_flux	Day_nr-WEIGHT-BLI_max_radiance	0.6971	0.0552
MRI_IPSI-MRI_CONTRA-BLI_max_flux-BLI_max_radiance	Day_nr-WEIGHT-NS	0.6947	0.0347
Day_nr-BLI_max_flux-BLI_max_radiance	MRI_IPSI-MRI_CONTRA-WEIGHT-NS	0.6917	0.0440
Day_nr-MRI_CONTRA-NS-BLI_max_flux	MRI_IPSI-WEIGHT-BLI_max_radiance	0.6917	0.0325
Day_nr-MRI_IPSI-WEIGHT-NS-BLI_max_flux	MRI_CONTRA-BLI_max_radiance	0.6840	0.0695
Day_nr-MRI_CONTRA-WEIGHT-NS-BLI_max_flux	MRI_IPSI-BLI_max_radiance	0.6822	0.0349
Day_nr-WEIGHT-NS-BLI_max_flux	MRI_IPSI-MRI_CONTRA-BLI_max_radiance	0.6790	0.0543
Day_nr-MRI_IPSI-NS-	MRI_CONTRA-WEIGHT-	0.6735	0.0421

BLI_max_radiance	BLI_max_flux		
Day_nr-NS-BLI_max_flux	MRI_IPSI-MRI_CONTRA-WEIGHT-BLI_max_radiance	0.6709	0.0380
MRI_IPSI-NS-BLI_max_flux	Day_nr-MRI_CONTRA-WEIGHT-BLI_max_radiance	0.6697	0.0513
Day_nr-MRI_IPSI-BLI_max_flux	MRI_CONTRA-WEIGHT-NS-BLI_max_radiance	0.6678	0.0312
MRI_CONTRA-NS-BLI_max_flux-BLI_max_radiance	Day_nr-MRI_IPSI-WEIGHT	0.6628	0.0301
NS-BLI_max_flux-BLI_max_radiance	Day_nr-MRI_IPSI-MRI_CONTRA-WEIGHT	0.6581	0.0338
Day_nr-MRI_IPSI-WEIGHT-BLI_max_flux-BLI_max_radiance	MRI_CONTRA-NS	0.6528	0.0697
Day_nr-MRI_IPSI-NS-BLI_max_flux	MRI_CONTRA-WEIGHT-BLI_max_radiance	0.6455	0.0318
Day_nr-MRI_IPSI-BLI_max_flux-BLI_max_radiance	MRI_CONTRA-WEIGHT-NS	0.6434	0.0324
Day_nr-MRI_IPSI-MRI_CONTRA-NS-BLI_max_flux	WEIGHT-BLI_max_radiance	0.6391	0.0312
Day_nr-MRI_CONTRA-NS-BLI_max_flux-BLI_max_radiance	MRI_IPSI-WEIGHT	0.6370	0.0405
Day_nr-MRI_IPSI-MRI_CONTRA-BLI_max_flux-BLI_max_radiance	WEIGHT-NS	0.6342	0.0361
Day_nr-NS-BLI_max_flux-BLI_max_radiance	MRI_IPSI-MRI_CONTRA-WEIGHT	0.6336	0.0342
Day_nr-MRI_CONTRA-WEIGHT-NS-BLI_max_flux-	MRI_IPSI	0.6297	0.0355

BLI_max_radiance			
MRI_IPSI-NS-BLI_max_flux-BLI_max_radiance	Day_nr-MRI_CONTRA-WEIGHT	0.6263	0.0339
Day_nr-MRI_IPSI-NS-BLI_max_flux-BLI_max_radiance	MRI_CONTRA-WEIGHT	0.6234	0.0304
MRI_CONTRA-WEIGHT-NS-BLI_max_flux-BLI_max_radiance	Day_nr-MRI_IPSI	0.6228	0.0289
MRI_IPSI-MRI_CONTRA-NS-BLI_max_flux-BLI_max_radiance	Day_nr-WEIGHT	0.6023	0.0188
WEIGHT-NS-BLI_max_flux-BLI_max_radiance	Day_nr-MRI_IPSI-MRI_CONTRA	0.5983	0.0441
Day_nr-WEIGHT-NS-BLI_max_flux-BLI_max_radiance	MRI_IPSI-MRI_CONTRA	0.5846	0.0350
Day_nr-MRI_IPSI-MRI_CONTRA-NS-BLI_max_flux-BLI_max_radiance	WEIGHT	0.5824	0.0152
MRI_IPSI-WEIGHT-NS-BLI_max_flux-BLI_max_radiance	Day_nr-MRI_CONTRA	0.5786	0.0183
Day_nr-MRI_IPSI-WEIGHT-NS-BLI_max_flux-BLI_max_radiance	MRI_CONTRA	0.5766	0.0154
MRI_IPSI-MRI_CONTRA-WEIGHT-NS-BLI_max_flux-BLI_max_radiance	Day_nr	0.3136	0.2362

ANN - artificial neural network; SPL-sham - sham-operated mice group; SD - standard deviation; MRI_CONTRA - volume of the contralateral hemisphere measured by MRI; MRI_IPSI - volume of the ipsilateral hemisphere measured by MRI; BLI_max_radiance - area of peak radiation measured by bioluminescence method; BLI_max_flux - area of peak growth

measured by bioluminescence method; WEIGHT - animal weight; Day_nr - day from the middle carotid artery occlusion (MCAO) procedure; NS - scoring of phenotypic neurological assessment.
