# Supplementary material for: Innovative statistical approaches: the use of neural networks reduces the sample size in the splenectomy-MCAO mouse model
Source: Croat Med J. 2024 Apr;65(2):122–37. doi: 10.3325/cmj.2024.65.122 (PMC11074938; doi:10.3325/cmj.2024.65.122)
Supplement: Supplementary Table 3 [file CroatMedJ_65_s003.pdf]

Supplemental Table 3. Differences in prediction accuracies of ANN classes SPLX and SPL-sham depending on the exclusion of variables and their combinations, along with the results of the t-test. The ANN was trained using a dataset containing all days after the stroke. Values in the table are sorted based on the differences in mean accuracy predictions of classes SPLX and SPL-sham.

"Out" variable	"In" variable	Mean accuracy of SPLX – Mean accuracy of SPL-sham	t-test P-value	Statistically significant difference in the prediction of SPLX and SPL-sham
Day_nr-MRI_IPSI-MRI_CONTRA-WEIGHT-BLI_max_flux-BLI_max_radiance	NS	0.1742	0.0000	YES
MRI_IPSI-MRI_CONTRA-WEIGHT-NS-BLI_max_flux	Day_nr-BLI_max_radiance	0.1565	0.0000	YES
Day_nr-MRI_IPSI-MRI_CONTRA-WEIGHT-NS-BLI_max_flux	BLI_max_radiance	0.1539	0.0000	YES
Day_nr-MRI_IPSI-MRI_CONTRA-WEIGHT-BLI_max_radiance	NS-BLI_max_flux	0.1332	0.0000	YES
Day_nr-MRI_IPSI-MRI_CONTRA-WEIGHT-NS-BLI_max_radiance	BLI_max_flux	0.1247	0.0000	YES
MRI_IPSI-MRI_CONTRA-WEIGHT-BLI_max_flux	Day_nr-NS-BLI_max_radiance	0.1200	0.0000	YES
Day_nr-MRI_IPSI-WEIGHT-BLI_max_radiance	MRI_CONTRA-NS-BLI_max_flux	0.1181	0.0000	YES
MRI_CONTRA-WEIGHT-NS-BLI_max_flux	Day_nr-MRI_IPSI-BLI_max_radiance	0.1167	0.0000	YES
Day_nr-MRI_IPSI-MRI_CONTRA-BLI_max_radiance	WEIGHT-NS-BLI_max_flux	0.1162	0.0000	YES

Day_nr-MRI_IPSI-MRI_CONTRA-WEIGHT-BLI_max_flux	NS-BLI_max_radiance	0.1089	0.0000	YES
MRI_IPSI-MRI_CONTRA-WEIGHT-NS	Day_nr-BLI_max_flux-BLI_max_radiance	0.1040	0.0000	YES
MRI_IPSI-MRI_CONTRA-WEIGHT-BLI_max_flux-BLI_max_radiance	Day_nr-NS	0.1027	0.0000	YES
MRI_IPSI-WEIGHT-NS-BLI_max_flux	Day_nr-MRI_CONTRA-BLI_max_radiance	0.1005	0.0000	YES
Day_nr-MRI_IPSI-MRI_CONTRA-WEIGHT	NS-BLI_max_flux-BLI_max_radiance	0.0981	0.0000	YES
MRI_IPSI-MRI_CONTRA-WEIGHT-NS-BLI_max_radiance	Day_nr-BLI_max_flux	0.0964	0.0000	YES
MRI_IPSI-MRI_CONTRA-WEIGHT	Day_nr-NS-BLI_max_flux-BLI_max_radiance	0.0942	0.0000	YES
Day_nr-MRI_IPSI-WEIGHT	MRI_CONTRA-NS-BLI_max_flux-BLI_max_radiance	0.0935	0.0000	YES
MRI_CONTRA-WEIGHT-BLI_max_flux	Day_nr-MRI_IPSI-NS-BLI_max_radiance	0.0935	0.0000	YES
MRI_CONTRA-WEIGHT-NS	Day_nr-MRI_IPSI-BLI_max_flux-BLI_max_radiance	0.0857	0.0000	YES
Day_nr-MRI_IPSI-MRI_CONTRA-WEIGHT-NS	BLI_max_flux-BLI_max_radiance	0.0800	0.0000	YES
MRI_CONTRA-WEIGHT-BLI_max_radiance	Day_nr-MRI_IPSI-NS-BLI_max_flux	0.0795	0.0000	YES
Day_nr-MRI_CONTRA-WEIGHT-NS-BLI_max_flux	MRI_IPSI-BLI_max_radiance	0.0749	0.0000	YES
WEIGHT-NS-BLI_max_flux	Day_nr-MRI_IPSI-MRI_CONTRA-	0.0743	0.0000	YES

	BLI_max_radiance			
MRI_IPSI-WEIGHT-NS	Day_nr-MRI_CONTRA-BLI_max_flux-BLI_max_radiance	0.0739	0.0000	YES
Day_nr-MRI_IPSI-MRI_CONTRA	WEIGHT-NS-BLI_max_flux-BLI_max_radiance	0.0722	0.0000	YES
MRI_IPSI-MRI_CONTRA-WEIGHT-BLI_max_radiance	Day_nr-NS-BLI_max_flux	0.0716	0.0000	YES
Day_nr-MRI_CONTRA-WEIGHT-NS-BLI_max_flux-BLI_max_radiance	MRI_IPSI	0.0710	0.0000	YES
MRI_CONTRA-WEIGHT	Day_nr-MRI_IPSI-NS-BLI_max_flux-BLI_max_radiance	0.0710	0.0000	YES
Day_nr-MRI_IPSI-WEIGHT-NS	MRI_CONTRA-BLI_max_flux-BLI_max_radiance	0.0691	0.0000	YES
Day_nr-MRI_CONTRA-WEIGHT-BLI_max_flux-BLI_max_radiance	MRI_IPSI-NS	0.0687	0.0000	YES
Day_nr-MRI_IPSI-MRI_CONTRA-NS-BLI_max_radiance	WEIGHT-BLI_max_flux	0.0686	0.0000	YES
Day_nr-MRI_CONTRA-WEIGHT-BLI_max_flux	MRI_IPSI-NS-BLI_max_radiance	0.0685	0.0000	YES
Day_nr-MRI_IPSI-WEIGHT-BLI_max_flux	MRI_CONTRA-NS-BLI_max_radiance	0.0675	0.0000	YES
MRI_CONTRA-WEIGHT-BLI_max_flux-BLI_max_radiance	Day_nr-MRI_IPSI-NS	0.0663	0.0000	YES
MRI_CONTRA-NS-BLI_max_flux	Day_nr-MRI_IPSI-WEIGHT-BLI_max_radiance	0.0646	0.0000	YES

MRI_IPSI-MRI_CONTRA-BLI_max_flux	Day_nr-WEIGHT-NS-BLI_max_radiance	0.0632	0.0000	YES
MRI_IPSI-WEIGHT-BLI_max_flux	Day_nr-MRI_CONTRA-NS-BLI_max_radiance	0.0624	0.0000	YES
MRI_IPSI-WEIGHT	Day_nr-MRI_CONTRA-NS-BLI_max_flux-BLI_max_radiance	0.0612	0.0000	YES
Day_nr-MRI_IPSI-WEIGHT-NS-BLI_max_radiance	MRI_CONTRA-BLI_max_flux	0.0612	0.0001	YES
Day_nr-MRI_CONTRA-WEIGHT-BLI_max_radiance	MRI_IPSI-NS-BLI_max_flux	0.0569	0.0000	YES
Day_nr-MRI_IPSI-MRI_CONTRA-NS	WEIGHT-BLI_max_flux-BLI_max_radiance	0.0558	0.0000	YES
Day_nr-MRI_IPSI-MRI_CONTRA-BLI_max_flux	WEIGHT-NS-BLI_max_radiance	0.0546	0.0000	YES
WEIGHT-NS	Day_nr-MRI_IPSI-MRI_CONTRA-BLI_max_flux-BLI_max_radiance	0.0544	0.0001	YES
WEIGHT-BLI_max_flux	Day_nr-MRI_IPSI-MRI_CONTRA-NS-BLI_max_radiance	0.0528	0.0000	YES
MRI_CONTRA-WEIGHT-NS-BLI_max_radiance	Day_nr-MRI_IPSI-BLI_max_flux	0.0526	0.0000	YES
MRI_IPSI-MRI_CONTRA	Day_nr-WEIGHT-NS-BLI_max_flux-BLI_max_radiance	0.0522	0.0000	YES
MRI_IPSI-MRI_CONTRA-NS	Day_nr-WEIGHT-BLI_max_flux-BLI_max_radiance	0.0516	0.0000	YES
MRI_CONTRA-WEIGHT-NS-BLI_max_flux-BLI_max_radiance	Day_nr-MRI_IPSI	0.0514	0.0000	YES

Day_nr-MRI_CONTRA-WEIGHT-NS-BLI_max_radiance	MRI_IPSI-BLI_max_flux	0.0492	0.0000	YES
MRI_IPSI-BLI_max_flux	Day_nr-MRI_CONTRA-WEIGHT-NS-BLI_max_radiance	0.0483	0.0000	YES
Day_nr-MRI_CONTRA-WEIGHT	MRI_IPSI-NS-BLI_max_flux-BLI_max_radiance	0.0469	0.0000	YES
Day_nr-MRI_CONTRA-WEIGHT-NS	MRI_IPSI-BLI_max_flux-BLI_max_radiance	0.0468	0.0001	YES
Day_nr-MRI_CONTRA-BLI_max_radiance	MRI_IPSI-WEIGHT-NS-BLI_max_flux	0.0439	0.0002	YES
Day_nr-WEIGHT-BLI_max_radiance	MRI_IPSI-MRI_CONTRA-NS-BLI_max_flux	0.0439	0.0000	YES
MRI_IPSI-MRI_CONTRA-BLI_max_radiance	Day_nr-WEIGHT-NS-BLI_max_flux	0.0433	0.0000	YES
MRI_IPSI-NS	Day_nr-MRI_CONTRA-WEIGHT-BLI_max_flux-BLI_max_radiance	0.0429	0.0001	YES
MRI_IPSI-MRI_CONTRA-NS-BLI_max_radiance	Day_nr-WEIGHT-BLI_max_flux	0.0429	0.0000	YES
WEIGHT	Day_nr-MRI_IPSI-MRI_CONTRA-NS-BLI_max_flux-BLI_max_radiance	0.0420	0.0000	YES
Day_nr-MRI_CONTRA-BLI_max_flux	MRI_IPSI-WEIGHT-NS-BLI_max_radiance	0.0402	0.0005	YES
Day_nr-WEIGHT-BLI_max_flux	MRI_IPSI-MRI_CONTRA-NS-BLI_max_radiance	0.0400	0.0005	YES
MRI_IPSI-WEIGHT-BLI_max_flux-BLI_max_radiance	Day_nr-MRI_CONTRA-NS	0.0398	0.0002	YES
MRI_IPSI-WEIGHT-	Day_nr-MRI_CONTRA-NS-	0.0383	0.0001	YES

BLI_max_radiance	BLI_max_flux			
MRI_IPSI	Day_nr-MRI_CONTRA-WEIGHT-NS-BLI_max_flux-BLI_max_radiance	0.0376	0.0001	YES
Day_nr-WEIGHT	MRI_IPSI-MRI_CONTRA-NS-BLI_max_flux-BLI_max_radiance	0.0369	0.0002	YES
MRI_CONTRA-BLI_max_flux	Day_nr-MRI_IPSI-WEIGHT-NS-BLI_max_radiance	0.0366	0.0024	YES
MRI_IPSI-NS-BLI_max_radiance	Day_nr-MRI_CONTRA-WEIGHT-BLI_max_flux	0.0360	0.0006	YES
MRI_IPSI-MRI_CONTRA-NS-BLI_max_flux	Day_nr-WEIGHT-BLI_max_radiance	0.0351	0.0358	YES
Day_nr-MRI_CONTRA	MRI_IPSI-WEIGHT-NS-BLI_max_flux-BLI_max_radiance	0.0348	0.0017	YES
MRI_IPSI-BLI_max_radiance	Day_nr-MRI_CONTRA-WEIGHT-NS-BLI_max_flux	0.0333	0.0002	YES
Day_nr-MRI_CONTRA-NS-BLI_max_radiance	MRI_IPSI-WEIGHT-BLI_max_flux	0.0333	0.0020	YES
NS	Day_nr-MRI_IPSI-MRI_CONTRA-WEIGHT-BLI_max_flux-BLI_max_radiance	0.0325	0.0023	YES
MRI_IPSI-MRI_CONTRA-BLI_max_flux-BLI_max_radiance	Day_nr-WEIGHT-NS	0.0319	0.0025	YES
Day_nr-WEIGHT-NS-BLI_max_radiance	MRI_IPSI-MRI_CONTRA-BLI_max_flux	0.0300	0.0049	YES
MRI_CONTRA	Day_nr-MRI_IPSI-WEIGHT-NS-BLI_max_flux-BLI_max_radiance	0.0285	0.0017	YES

Day_nr-WEIGHT-BLI_max_flux-BLI_max_radiance	MRI_IPSI-MRI_CONTRA-NS	0.0281	0.0136	YES
WEIGHT-BLI_max_radiance	Day_nr-MRI_IPSI-MRI_CONTRA-NS-BLI_max_flux	0.0278	0.0056	YES
Day_nr-WEIGHT-NS	MRI_IPSI-MRI_CONTRA-BLI_max_flux-BLI_max_radiance	0.0273	0.0126	YES
NS-BLI_max_flux	Day_nr-MRI_IPSI-MRI_CONTRA-WEIGHT-BLI_max_radiance	0.0269	0.0231	YES
BLI_max_flux	Day_nr-MRI_IPSI-MRI_CONTRA-WEIGHT-NS-BLI_max_radiance	0.0267	0.0214	YES
MRI_CONTRA-NS	Day_nr-MRI_IPSI-WEIGHT-BLI_max_flux-BLI_max_radiance	0.0267	0.0104	YES
None	Day_nr-MRI_IPSI-MRI_CONTRA-WEIGHT-NS-BLI_max_flux-BLI_max_radiance	0.0264	0.0018	YES
Day_nr-MRI_CONTRA-NS	MRI_IPSI-WEIGHT-BLI_max_flux-BLI_max_radiance	0.0255	0.0196	YES
Day_nr-MRI_IPSI-WEIGHT-BLI_max_flux-BLI_max_radiance	MRI_CONTRA-NS	0.0241	0.1912	NO
BLI_max_radiance	Day_nr-MRI_IPSI-MRI_CONTRA-WEIGHT-NS-BLI_max_flux	0.0235	0.0099	YES
Day_nr-MRI_CONTRA-BLI_max_flux-BLI_max_radiance	MRI_IPSI-WEIGHT-NS	0.0234	0.0222	YES
MRI_CONTRA-BLI_max_flux-	Day_nr-MRI_IPSI-WEIGHT-NS	0.0233	0.0176	YES

BLI_max_radiance				
Day_nr-BLI_max_radiance	MRI_IPSI-MRI_CONTRA-WEIGHT-NS-BLI_max_flux	0.0233	0.0180	YES
MRI_CONTRA-BLI_max_radiance	Day_nr-MRI_IPSI-WEIGHT-NS-BLI_max_flux	0.0226	0.0120	YES
Day_nr	MRI_IPSI-MRI_CONTRA-WEIGHT-NS-BLI_max_flux-BLI_max_radiance	0.0219	0.0567	NO
WEIGHT-NS-BLI_max_radiance	Day_nr-MRI_IPSI-MRI_CONTRA-BLI_max_flux	0.0217	0.0660	NO
MRI_IPSI-WEIGHT-NS-BLI_max_radiance	Day_nr-MRI_CONTRA-BLI_max_flux	0.0216	0.0398	YES
MRI_CONTRA-NS-BLI_max_radiance	Day_nr-MRI_IPSI-WEIGHT-BLI_max_flux	0.0203	0.0459	YES
WEIGHT-BLI_max_flux-BLI_max_radiance	Day_nr-MRI_IPSI-MRI_CONTRA-NS	0.0199	0.0796	NO
Day_nr-WEIGHT-NS-BLI_max_flux	MRI_IPSI-MRI_CONTRA-BLI_max_radiance	0.0175	0.2589	NO
Day_nr-MRI_IPSI	MRI_CONTRA-WEIGHT-NS-BLI_max_flux-BLI_max_radiance	0.0142	0.3633	NO
NS-BLI_max_radiance	Day_nr-MRI_IPSI-MRI_CONTRA-WEIGHT-BLI_max_flux	0.0141	0.1297	NO
Day_nr-MRI_CONTRA-NS-BLI_max_flux	MRI_IPSI-WEIGHT-BLI_max_radiance	0.0111	0.3201	NO
Day_nr-NS-BLI_max_radiance	MRI_IPSI-MRI_CONTRA-WEIGHT-BLI_max_flux	0.0083	0.4498	NO
Day_nr-NS	MRI_IPSI-MRI_CONTRA-WEIGHT-BLI_max_flux-BLI_max_radiance	0.0077	0.4404	NO
Day_nr-BLI_max_flux	MRI_IPSI-MRI_CONTRA-	0.0038	0.7368	NO

	WEIGHT-NS-BLI_max_radiance			
BLI_max_flux-BLI_max_radiance	Day_nr-MRI_IPSI-MRI_CONTRA-WEIGHT-NS	-0.0032	0.7473	NO
MRI_IPSI-BLI_max_flux-BLI_max_radiance	Day_nr-MRI_CONTRA-WEIGHT-NS	-0.0067	0.5194	NO
MRI_CONTRA-NS-BLI_max_flux-BLI_max_radiance	Day_nr-MRI_IPSI-WEIGHT	-0.0091	0.3229	NO
Day_nr-MRI_IPSI-BLI_max_radiance	MRI_CONTRA-WEIGHT-NS-BLI_max_flux	-0.0136	0.4909	NO
Day_nr-MRI_IPSI-WEIGHT-NS-BLI_max_flux	MRI_CONTRA-BLI_max_radiance	-0.0212	0.2652	NO
Day_nr-MRI_IPSI-NS	MRI_CONTRA-WEIGHT-BLI_max_flux-BLI_max_radiance	-0.0293	0.0678	NO
Day_nr-MRI_IPSI-MRI_CONTRA-BLI_max_flux-BLI_max_radiance	WEIGHT-NS	-0.0358	0.0446	YES
Day_nr-BLI_max_flux-BLI_max_radiance	MRI_IPSI-MRI_CONTRA-WEIGHT-NS	-0.0398	0.0015	YES
Day_nr-MRI_IPSI-NS-BLI_max_radiance	MRI_CONTRA-WEIGHT-BLI_max_flux	-0.0458	0.0041	YES
MRI_IPSI-NS-BLI_max_flux	Day_nr-MRI_CONTRA-WEIGHT-BLI_max_radiance	-0.0477	0.0074	YES
Day_nr-MRI_CONTRA-NS-BLI_max_flux-BLI_max_radiance	MRI_IPSI-WEIGHT	-0.0496	0.0003	YES
NS-BLI_max_flux-BLI_max_radiance	Day_nr-MRI_IPSI-MRI_CONTRA-WEIGHT	-0.0522	0.0000	YES
Day_nr-NS-BLI_max_flux	MRI_IPSI-MRI_CONTRA-	-0.0542	0.0007	YES

	WEIGHT-BLI_max_radiance			
MRI_IPSI-MRI_CONTRA-NS-BLI_max_flux-BLI_max_radiance	Day_nr-WEIGHT	-0.0616	0.0000	YES
WEIGHT-NS-BLI_max_flux-BLI_max_radiance	Day_nr-MRI_IPSI-MRI_CONTRA	-0.0683	0.0000	YES
Day_nr-MRI_IPSI-MRI_CONTRA-NS-BLI_max_flux-BLI_max_radiance	WEIGHT	-0.0785	0.0000	YES
Day_nr-MRI_IPSI-MRI_CONTRA-NS-BLI_max_flux	WEIGHT-BLI_max_radiance	-0.0808	0.0000	YES
Day_nr-MRI_IPSI-BLI_max_flux	MRI_CONTRA-WEIGHT-NS-BLI_max_radiance	-0.0856	0.0000	YES
MRI_IPSI-MRI_CONTRA-WEIGHT-NS-BLI_max_flux-BLI_max_radiance	Day_nr	-0.0998	0.1403	NO
Day_nr-NS-BLI_max_flux-BLI_max_radiance	MRI_IPSI-MRI_CONTRA-WEIGHT	-0.1015	0.0000	YES
Day_nr-MRI_IPSI-BLI_max_flux-BLI_max_radiance	MRI_CONTRA-WEIGHT-NS	-0.1051	0.0000	YES
MRI_IPSI-NS-BLI_max_flux-BLI_max_radiance	Day_nr-MRI_CONTRA-WEIGHT	-0.1065	0.0000	YES
Day_nr-WEIGHT-NS-BLI_max_flux-BLI_max_radiance	MRI_IPSI-MRI_CONTRA	-0.1102	0.0000	YES
Day_nr-MRI_IPSI-NS-BLI_max_flux	MRI_CONTRA-WEIGHT-BLI_max_radiance	-0.1112	0.0000	YES
Day_nr-MRI_IPSI-NS-	MRI_CONTRA-WEIGHT	-0.1113	0.0000	YES

BLI_max_flux-BLI_max_radiance				
MRI_IPSI-WEIGHT-NS-BLI_max_flux-BLI_max_radiance	Day_nr-MRI_CONTRA	-0.1124	0.0000	YES
Day_nr-MRI_IPSI-WEIGHT-NS-BLI_max_flux-BLI_max_radiance	MRI_CONTRA	-0.1237	0.0000	YES

ANN - artificial neural network; SPLX - splenectomized mice group; SPL-sham - sham-operated mice group; SD - standard deviation; MRI_CONTRA - volume of contralateral hemisphere measured by MRI; MRI_IPSI - volume of ipsilateral hemisphere measured by MRI; BLI_max_radiance - surface area of peak radiation measured by bioluminescence method; BLI_max_flux - surface area of peak growth measured by bioluminescence method; WEIGHT - animal weight; Day_nr - day from the middle carotid artery occlusion (MCAO) procedure; NS - scoring of phenotypic neurological assessment.
