# Supplementary material for: Innovative statistical approaches: the use of neural networks reduces the sample size in the splenectomy-MCAO mouse model
Source: Croat Med J. 2024 Apr;65(2):122–37. doi: 10.3325/cmj.2024.65.122 (PMC11074938; doi:10.3325/cmj.2024.65.122)
Supplement: Supplementary Table 4 [file CroatMedJ_65_s004.pdf]

Supplemental Table 4. Prediction accuracy of ANN class SPLX depending on the exclusion of variables and their combinations. The ANN was trained using a dataset containing all days after the stroke, except the 2nd day. Values in the table are sorted based on the mean accuracy prediction of class SPLX.

"Out" variable	"In" variable	The mean accuracy value of ANN predictions for the SPLX class.	SD of accuracy in predictions for the ANN SPLX class.
None	Day_nr-MRI_IPSI-MRI_CONTRA-WEIGHT-NS-BLI_max_flux-BLI_max_radiance	0.8485	0.0254
MRI_IPSI	Day_nr-MRI_CONTRA-WEIGHT-NS-BLI_max_flux-BLI_max_radiance	0.8474	0.0214
MRI_CONTRA	Day_nr-MRI_IPSI-WEIGHT-NS-BLI_max_flux-BLI_max_radiance	0.8459	0.0257
MRI_IPSI-MRI_CONTRA	Day_nr-WEIGHT-NS-BLI_max_flux-BLI_max_radiance	0.8360	0.0253
NS	Day_nr-MRI_IPSI-MRI_CONTRA-WEIGHT-BLI_max_flux-BLI_max_radiance	0.8334	0.0317
WEIGHT	Day_nr-MRI_IPSI-MRI_CONTRA-NS-BLI_max_flux-BLI_max_radiance	0.8319	0.0284
MRI_IPSI-WEIGHT	Day_nr-MRI_CONTRA-NS-BLI_max_flux-BLI_max_radiance	0.8300	0.0218
MRI_IPSI-NS	Day_nr-MRI_CONTRA-	0.8289	0.0281

	WEIGHT-BLI_max_flux-BLI_max_radiance		
MRI_CONTRA-NS	Day_nr-MRI_IPSI-WEIGHT-BLI_max_flux-BLI_max_radiance	0.8274	0.0280
MRI_IPSI-MRI_CONTRA-NS	Day_nr-WEIGHT-BLI_max_flux-BLI_max_radiance	0.8203	0.0244
MRI_CONTRA-WEIGHT	Day_nr-MRI_IPSI-NS-BLI_max_flux-BLI_max_radiance	0.8160	0.0244
MRI_IPSI-BLI_max_radiance	Day_nr-MRI_CONTRA-WEIGHT-NS-BLI_max_flux	0.8159	0.0275
BLI_max_radiance	Day_nr-MRI_IPSI-MRI_CONTRA-WEIGHT-NS-BLI_max_flux	0.8158	0.0224
BLI_max_flux	Day_nr-MRI_IPSI-MRI_CONTRA-WEIGHT-NS-BLI_max_radiance	0.8125	0.0341
MRI_IPSI-MRI_CONTRA-WEIGHT	Day_nr-NS-BLI_max_flux-BLI_max_radiance	0.8124	0.0221
MRI_CONTRA-BLI_max_flux	Day_nr-MRI_IPSI-WEIGHT-NS-BLI_max_radiance	0.8096	0.0302
MRI_IPSI-MRI_CONTRA-BLI_max_radiance	Day_nr-WEIGHT-NS-BLI_max_flux	0.8084	0.0258
NS-BLI_max_radiance	Day_nr-MRI_IPSI-MRI_CONTRA-WEIGHT-BLI_max_flux	0.8057	0.0263
MRI_IPSI-BLI_max_flux	Day_nr-MRI_CONTRA-WEIGHT-NS-BLI_max_radiance	0.8023	0.0308
MRI_CONTRA-BLI_max_radiance	Day_nr-MRI_IPSI-WEIGHT-NS-BLI_max_flux	0.7990	0.0271
WEIGHT-NS	Day_nr-MRI_IPSI-	0.7985	0.0288

	MRI_CONTRA-BLI_max_flux-BLI_max_radiance		
MRI_IPSI-NS-BLI_max_radiance	Day_nr-MRI_CONTRA-WEIGHT-BLI_max_flux	0.7982	0.0283
MRI_IPSI-WEIGHT-NS	Day_nr-MRI_CONTRA-BLI_max_flux-BLI_max_radiance	0.7936	0.0208
MRI_CONTRA-NS-BLI_max_radiance	Day_nr-MRI_IPSI-WEIGHT-BLI_max_flux	0.7930	0.0325
MRI_IPSI-MRI_CONTRA-BLI_max_flux	Day_nr-WEIGHT-NS-BLI_max_radiance	0.7924	0.0334
WEIGHT-BLI_max_radiance	Day_nr-MRI_IPSI-MRI_CONTRA-NS-BLI_max_flux	0.7910	0.0268
MRI_CONTRA-WEIGHT-NS	Day_nr-MRI_IPSI-BLI_max_flux-BLI_max_radiance	0.7890	0.0264
MRI_IPSI-WEIGHT-BLI_max_radiance	Day_nr-MRI_CONTRA-NS-BLI_max_flux	0.7872	0.0204
Day_nr-BLI_max_radiance	MRI_IPSI-MRI_CONTRA-WEIGHT-NS-BLI_max_flux	0.7848	0.0313
MRI_IPSI-MRI_CONTRA-WEIGHT-NS	Day_nr-BLI_max_flux-BLI_max_radiance	0.7848	0.0251
NS-BLI_max_flux	Day_nr-MRI_IPSI-MRI_CONTRA-WEIGHT-BLI_max_radiance	0.7847	0.0359
Day_nr	MRI_IPSI-MRI_CONTRA-WEIGHT-NS-BLI_max_flux-BLI_max_radiance	0.7843	0.0241
MRI_IPSI-MRI_CONTRA-NS-BLI_max_radiance	Day_nr-WEIGHT-BLI_max_flux	0.7802	0.0280
Day_nr-MRI_IPSI-NS-BLI_max_flux	MRI_CONTRA-WEIGHT-BLI_max_radiance	0.7759	0.0335

Day_nr-NS	MRI_IPSI-MRI_CONTRA-WEIGHT-BLI_max_flux-BLI_max_radiance	0.7746	0.0262
Day_nr-MRI_IPSI-BLI_max_flux	MRI_CONTRA-WEIGHT-NS-BLI_max_radiance	0.7745	0.0391
Day_nr-BLI_max_flux	MRI_IPSI-MRI_CONTRA-WEIGHT-NS-BLI_max_radiance	0.7714	0.0264
MRI_CONTRA-NS-BLI_max_flux	Day_nr-MRI_IPSI-WEIGHT-BLI_max_radiance	0.7711	0.0385
MRI_IPSI-WEIGHT-BLI_max_flux	Day_nr-MRI_CONTRA-NS-BLI_max_radiance	0.7702	0.0237
MRI_IPSI-NS-BLI_max_flux	Day_nr-MRI_CONTRA-WEIGHT-BLI_max_radiance	0.7700	0.0327
WEIGHT-BLI_max_flux	Day_nr-MRI_IPSI-MRI_CONTRA-NS-BLI_max_radiance	0.7697	0.0339
MRI_CONTRA-WEIGHT-BLI_max_flux	Day_nr-MRI_IPSI-NS-BLI_max_radiance	0.7689	0.0217
MRI_CONTRA-WEIGHT-NS-BLI_max_flux	Day_nr-MRI_IPSI-BLI_max_radiance	0.7676	0.0316
Day_nr-WEIGHT	MRI_IPSI-MRI_CONTRA-NS-BLI_max_flux-BLI_max_radiance	0.7650	0.0227
Day_nr-BLI_max_flux-BLI_max_radiance	MRI_IPSI-MRI_CONTRA-WEIGHT-NS	0.7648	0.0432
Day_nr-MRI_CONTRA	MRI_IPSI-WEIGHT-NS-BLI_max_flux-BLI_max_radiance	0.7638	0.0265
BLI_max_flux-BLI_max_radiance	Day_nr-MRI_IPSI-MRI_CONTRA-WEIGHT-NS	0.7610	0.0352
Day_nr-NS-BLI_max_radiance	MRI_IPSI-MRI_CONTRA-WEIGHT-BLI_max_flux	0.7604	0.0312
Day_nr-MRI_CONTRA-	MRI_IPSI-WEIGHT-NS-	0.7584	0.0252

BLI_max_radiance	BLI_max_flux		
MRI_IPSI-MRI_CONTRA-WEIGHT-BLI_max_flux	Day_nr-NS-BLI_max_radiance	0.7582	0.0254
WEIGHT-NS-BLI_max_flux	Day_nr-MRI_IPSI-MRI_CONTRA-BLI_max_radiance	0.7556	0.0617
Day_nr-MRI_IPSI-BLI_max_flux-BLI_max_radiance	MRI_CONTRA-WEIGHT-NS	0.7553	0.0323
Day_nr-MRI_CONTRA-NS	MRI_IPSI-WEIGHT-BLI_max_flux-BLI_max_radiance	0.7552	0.0283
Day_nr-NS-BLI_max_flux	MRI_IPSI-MRI_CONTRA-WEIGHT-BLI_max_radiance	0.7521	0.0292
WEIGHT-NS-BLI_max_radiance	Day_nr-MRI_IPSI-MRI_CONTRA-BLI_max_flux	0.7493	0.0309
MRI_IPSI-WEIGHT-NS-BLI_max_flux	Day_nr-MRI_CONTRA-BLI_max_radiance	0.7480	0.0365
Day_nr-WEIGHT-BLI_max_radiance	MRI_IPSI-MRI_CONTRA-NS-BLI_max_flux	0.7473	0.0328
MRI_IPSI-MRI_CONTRA-WEIGHT-BLI_max_radiance	Day_nr-NS-BLI_max_flux	0.7467	0.0274
Day_nr-MRI_IPSI-BLI_max_radiance	MRI_CONTRA-WEIGHT-NS-BLI_max_flux	0.7460	0.0547
Day_nr-MRI_IPSI-NS-BLI_max_radiance	MRI_CONTRA-WEIGHT-BLI_max_flux	0.7460	0.0523
Day_nr-MRI_IPSI-WEIGHT-NS-BLI_max_flux-BLI_max_radiance	MRI_CONTRA	0.7453	0.0355
MRI_CONTRA-WEIGHT-BLI_max_radiance	Day_nr-MRI_IPSI-NS-BLI_max_flux	0.7448	0.0281
MRI_IPSI-BLI_max_flux-	Day_nr-MRI_CONTRA-	0.7441	0.0330

BLI_max_radiance	WEIGHT-NS		
Day_nr-MRI_IPSI-NS-BLI_max_flux-BLI_max_radiance	MRI_CONTRA-WEIGHT	0.7424	0.0308
Day_nr-MRI_IPSI	MRI_CONTRA-WEIGHT-NS-BLI_max_flux-BLI_max_radiance	0.7408	0.0418
Day_nr-MRI_IPSI-NS	MRI_CONTRA-WEIGHT-BLI_max_flux-BLI_max_radiance	0.7406	0.0369
Day_nr-MRI_CONTRA-BLI_max_flux	MRI_IPSI-WEIGHT-NS-BLI_max_radiance	0.7401	0.0306
MRI_IPSI-WEIGHT-NS-BLI_max_radiance	Day_nr-MRI_CONTRA-BLI_max_flux	0.7400	0.0432
Day_nr-MRI_CONTRA-NS-BLI_max_radiance	MRI_IPSI-WEIGHT-BLI_max_flux	0.7388	0.0294
MRI_IPSI-MRI_CONTRA-NS-BLI_max_flux	Day_nr-WEIGHT-BLI_max_radiance	0.7341	0.0399
Day_nr-MRI_CONTRA-WEIGHT	MRI_IPSI-NS-BLI_max_flux-BLI_max_radiance	0.7338	0.0247
MRI_IPSI-MRI_CONTRA-WEIGHT-NS-BLI_max_flux	Day_nr-BLI_max_radiance	0.7330	0.0440
Day_nr-NS-BLI_max_flux-BLI_max_radiance	MRI_IPSI-MRI_CONTRA-WEIGHT	0.7328	0.0393
Day_nr-WEIGHT-NS	MRI_IPSI-MRI_CONTRA-BLI_max_flux-BLI_max_radiance	0.7316	0.0238
MRI_CONTRA-BLI_max_flux-BLI_max_radiance	Day_nr-MRI_IPSI-WEIGHT-NS	0.7310	0.0328
Day_nr-MRI_IPSI-WEIGHT-NS-BLI_max_flux	MRI_CONTRA-BLI_max_radiance	0.7276	0.0317
MRI_IPSI-NS-BLI_max_flux-	Day_nr-MRI_CONTRA-WEIGHT	0.7250	0.0283

BLI_max_radiance			
NS-BLI_max_flux-BLI_max_radiance	Day_nr-MRI_IPSI-MRI_CONTRA-WEIGHT	0.7245	0.0276
Day_nr-WEIGHT-BLI_max_flux	MRI_IPSI-MRI_CONTRA-NS-BLI_max_radiance	0.7227	0.0286
Day_nr-MRI_CONTRA-WEIGHT-BLI_max_radiance	MRI_IPSI-NS-BLI_max_flux	0.7218	0.0246
Day_nr-MRI_IPSI-MRI_CONTRA-NS-BLI_max_flux	WEIGHT-BLI_max_radiance	0.7204	0.0401
Day_nr-WEIGHT-NS-BLI_max_radiance	MRI_IPSI-MRI_CONTRA-BLI_max_flux	0.7199	0.0338
Day_nr-MRI_CONTRA-NS-BLI_max_flux	MRI_IPSI-WEIGHT-BLI_max_radiance	0.7197	0.0267
MRI_IPSI-WEIGHT-NS-BLI_max_flux-BLI_max_radiance	Day_nr-MRI_CONTRA	0.7169	0.0401
WEIGHT-BLI_max_flux-BLI_max_radiance	Day_nr-MRI_IPSI-MRI_CONTRA-NS	0.7167	0.0324
Day_nr-MRI_CONTRA-WEIGHT-NS	MRI_IPSI-BLI_max_flux-BLI_max_radiance	0.7119	0.0226
MRI_IPSI-MRI_CONTRA-BLI_max_flux-BLI_max_radiance	Day_nr-WEIGHT-NS	0.7103	0.0205
Day_nr-MRI_IPSI-MRI_CONTRA-NS-BLI_max_flux-BLI_max_radiance	WEIGHT	0.7064	0.0314
MRI_IPSI-MRI_CONTRA-WEIGHT-NS-BLI_max_radiance	Day_nr-BLI_max_flux	0.7047	0.0325
MRI_CONTRA-WEIGHT-NS-BLI_max_radiance	Day_nr-MRI_IPSI-BLI_max_flux	0.7042	0.0307

Day_nr-MRI_CONTRA-BLI_max_flux-BLI_max_radiance	MRI_IPSI-WEIGHT-NS	0.7030	0.0324
Day_nr-WEIGHT-BLI_max_flux-BLI_max_radiance	MRI_IPSI-MRI_CONTRA-NS	0.6991	0.0393
Day_nr-MRI_CONTRA-WEIGHT-BLI_max_flux	MRI_IPSI-NS-BLI_max_radiance	0.6975	0.0314
Day_nr-WEIGHT-NS-BLI_max_flux	MRI_IPSI-MRI_CONTRA-BLI_max_radiance	0.6913	0.0325
MRI_CONTRA-NS-BLI_max_flux-BLI_max_radiance	Day_nr-MRI_IPSI-WEIGHT	0.6913	0.0245
Day_nr-MRI_IPSI-MRI_CONTRA-NS-BLI_max_radiance	WEIGHT-BLI_max_flux	0.6889	0.0437
MRI_IPSI-WEIGHT-BLI_max_flux-BLI_max_radiance	Day_nr-MRI_CONTRA-NS	0.6873	0.0253
MRI_IPSI-MRI_CONTRA-NS-BLI_max_flux-BLI_max_radiance	Day_nr-WEIGHT	0.6821	0.0286
Day_nr-MRI_CONTRA-WEIGHT-NS-BLI_max_radiance	MRI_IPSI-BLI_max_flux	0.6812	0.0285
Day_nr-MRI_CONTRA-NS-BLI_max_flux-BLI_max_radiance	MRI_IPSI-WEIGHT	0.6807	0.0326
Day_nr-MRI_IPSI-MRI_CONTRA-NS	WEIGHT-BLI_max_flux-BLI_max_radiance	0.6787	0.0352
WEIGHT-NS-BLI_max_flux-BLI_max_radiance	Day_nr-MRI_IPSI-MRI_CONTRA	0.6776	0.0435
Day_nr-MRI_IPSI-MRI_CONTRA-BLI_max_flux-	WEIGHT-NS	0.6766	0.0604

BLI_max_radiance			
Day_nr-MRI_IPSI-WEIGHT-NS	MRI_CONTRA-BLI_max_flux-BLI_max_radiance	0.6719	0.0415
Day_nr-MRI_IPSI-WEIGHT-NS-BLI_max_radiance	MRI_CONTRA-BLI_max_flux	0.6714	0.0354
Day_nr-WEIGHT-NS-BLI_max_flux-BLI_max_radiance	MRI_IPSI-MRI_CONTRA	0.6707	0.0288
Day_nr-MRI_IPSI-MRI_CONTRA	WEIGHT-NS-BLI_max_flux-BLI_max_radiance	0.6704	0.0303
Day_nr-MRI_IPSI-WEIGHT	MRI_CONTRA-NS-BLI_max_flux-BLI_max_radiance	0.6679	0.0262
Day_nr-MRI_IPSI-MRI_CONTRA-BLI_max_flux	WEIGHT-NS-BLI_max_radiance	0.6641	0.0350
Day_nr-MRI_IPSI-MRI_CONTRA-BLI_max_radiance	WEIGHT-NS-BLI_max_flux	0.6639	0.0269
Day_nr-MRI_IPSI-WEIGHT-BLI_max_radiance	MRI_CONTRA-NS-BLI_max_flux	0.6635	0.0277
Day_nr-MRI_IPSI-WEIGHT-BLI_max_flux-BLI_max_radiance	MRI_CONTRA-NS	0.6612	0.0576
MRI_CONTRA-WEIGHT-BLI_max_flux-BLI_max_radiance	Day_nr-MRI_IPSI-NS	0.6606	0.0340
Day_nr-MRI_IPSI-MRI_CONTRA-WEIGHT-BLI_max_radiance	NS-BLI_max_flux	0.6592	0.0248
Day_nr-MRI_IPSI-WEIGHT-BLI_max_flux	MRI_CONTRA-NS-BLI_max_radiance	0.6587	0.0370
Day_nr-MRI_IPSI-	NS-BLI_max_flux-	0.6567	0.0236

MRI_CONTRA-WEIGHT	BLI_max_radiance		
Day_nr-MRI_CONTRA-WEIGHT-BLI_max_flux-BLI_max_radiance	MRI_IPSI-NS	0.6438	0.0300
Day_nr-MRI_IPSI-MRI_CONTRA-WEIGHT-NS	BLI_max_flux-BLI_max_radiance	0.6403	0.0270
Day_nr-MRI_CONTRA-WEIGHT-NS-BLI_max_flux	MRI_IPSI-BLI_max_radiance	0.6395	0.0353
MRI_IPSI-MRI_CONTRA-WEIGHT-BLI_max_flux-BLI_max_radiance	Day_nr-NS	0.6372	0.0236
Day_nr-MRI_IPSI-MRI_CONTRA-WEIGHT-BLI_max_flux	NS-BLI_max_radiance	0.6177	0.0225
Day_nr-MRI_IPSI-MRI_CONTRA-WEIGHT-NS-BLI_max_radiance	BLI_max_flux	0.6082	0.0247
Day_nr-MRI_IPSI-MRI_CONTRA-WEIGHT-BLI_max_flux-BLI_max_radiance	NS	0.5897	0.0199
MRI_CONTRA-WEIGHT-NS-BLI_max_flux-BLI_max_radiance	Day_nr-MRI_IPSI	0.5865	0.0340
Day_nr-MRI_CONTRA-WEIGHT-NS-BLI_max_flux-BLI_max_radiance	MRI_IPSI	0.5776	0.0377
Day_nr-MRI_IPSI-MRI_CONTRA-WEIGHT-NS-BLI_max_flux	BLI_max_radiance	0.5710	0.0309
MRI_IPSI-MRI_CONTRA-WEIGHT-NS-BLI_max_flux-BLI_max_radiance	Day_nr	0.3081	0.2327

ANN - artificial neural network; SPLX - splenectomized mice group; SD - standard deviation; MRI_CONTRA - volume of the contralateral hemisphere measured by MRI; MRI_IPSI - volume of the ipsilateral hemisphere measured by MRI; BLI_max_radiance - surface area of peak radiation measured by bioluminescence method; BLI_max_flux - surface area of peak growth measured by bioluminescence method; WEIGHT - animal weight; Day_nr - day from the middle carotid artery occlusion; MCAO procedure; NS - scoring of phenotypic neurological assessment.
