# Supplementary material for: Innovative statistical approaches: the use of neural networks reduces the sample size in the splenectomy-MCAO mouse model
Source: Croat Med J. 2024 Apr;65(2):122–37. doi: 10.3325/cmj.2024.65.122 (PMC11074938; doi:10.3325/cmj.2024.65.122)
Supplement: Supplementary Table 5 [file CroatMedJ_65_s005.pdf]

**Supplemental Table 5.** Prediction accuracy of ANN class SPL-sham depending on the exclusion of variables and their combinations. The ANN was trained using a dataset containing all days after the stroke, except the 2nd day. Values in the table are sorted based on the mean accuracy prediction of class SPL-sham.

| "Out" variable | "In" variable | The mean accuracy value of ANN predictions for the SPL-sham class. | SD of accuracy in predictions for the ANN SPL-sham class. |
|---|---|---|---|
| MRI_CONTRA-WEIGHT-NS | Day_nr-MRI_IPSI-BLI_max_flux-BLI_max_radiance | 0.9284 | 0.0366 |
| MRI_IPSI-MRI_CONTRA-WEIGHT-NS | Day_nr-BLI_max_flux-BLI_max_radiance | 0.9281 | 0.0286 |
| MRI_IPSI-MRI_CONTRA-WEIGHT | Day_nr-NS-BLI_max_flux-BLI_max_radiance | 0.9267 | 0.0213 |
| MRI_CONTRA-WEIGHT | Day_nr-MRI_IPSI-NS-BLI_max_flux-BLI_max_radiance | 0.9180 | 0.0385 |
| MRI_CONTRA-WEIGHT-NS-BLI_max_flux | Day_nr-MRI_IPSI-BLI_max_radiance | 0.9092 | 0.0321 |
| MRI_IPSI-WEIGHT | Day_nr-MRI_CONTRA-NS-BLI_max_flux-BLI_max_radiance | 0.9032 | 0.0347 |
| MRI_IPSI-MRI_CONTRA-WEIGHT-NS-BLI_max_flux | Day_nr-BLI_max_radiance | 0.8999 | 0.0303 |
| WEIGHT | Day_nr-MRI_IPSI-MRI_CONTRA-NS-BLI_max_flux-BLI_max_radiance | 0.8955 | 0.0340 |
| MRI_IPSI-MRI_CONTRA-WEIGHT-BLI_max_flux | Day_nr-NS-BLI_max_radiance | 0.8902 | 0.0337 |
| MRI_CONTRA-WEIGHT-BLI_max_flux | Day_nr-MRI_IPSI-NS-BLI_max_radiance | 0.8899 | 0.0343 |

| | | | |
|---|---|---|---|
| MRI_IPSI-WEIGHT-NS | Day_nr-MRI_CONTRA-BLI_max_flux-BLI_max_radiance | 0.8833 | 0.0442 |
| WEIGHT-NS | Day_nr-MRI_IPSI-MRI_CONTRA-BLI_max_flux-BLI_max_radiance | 0.8812 | 0.0492 |
| MRI_IPSI-MRI_CONTRA-NS | Day_nr-WEIGHT-BLI_max_flux-BLI_max_radiance | 0.8807 | 0.0336 |
| MRI_CONTRA | Day_nr-MRI_IPSI-WEIGHT-NS-BLI_max_flux-BLI_max_radiance | 0.8737 | 0.0300 |
| MRI_IPSI-NS | Day_nr-MRI_CONTRA-WEIGHT-BLI_max_flux-BLI_max_radiance | 0.8712 | 0.0340 |
| MRI_IPSI-MRI_CONTRA | Day_nr-WEIGHT-NS-BLI_max_flux-BLI_max_radiance | 0.8705 | 0.0355 |
| Day_nr-MRI_IPSI-MRI_CONTRA-WEIGHT-BLI_max_radiance | NS-BLI_max_flux | 0.8698 | 0.0300 |
| MRI_IPSI | Day_nr-MRI_CONTRA-WEIGHT-NS-BLI_max_flux-BLI_max_radiance | 0.8678 | 0.0242 |
| Day_nr-MRI_IPSI-WEIGHT-BLI_max_radiance | MRI_CONTRA-NS-BLI_max_flux | 0.8669 | 0.0338 |
| NS | Day_nr-MRI_IPSI-MRI_CONTRA-WEIGHT-BLI_max_flux-BLI_max_radiance | 0.8645 | 0.0387 |
| MRI_CONTRA-NS | Day_nr-MRI_IPSI-WEIGHT-BLI_max_flux-BLI_max_radiance | 0.8638 | 0.0304 |
| None | Day_nr-MRI_IPSI-MRI_CONTRA-WEIGHT- | 0.8576 | 0.0339 |

| | | | |
|---|---|---|---|
| | NS-BLI_max_flux-BLI_max_radiance | | |
| Day_nr-MRI_IPSI-MRI_CONTRA-BLI_max_radiance | WEIGHT-NS-BLI_max_flux | 0.8461 | 0.0317 |
| MRI_IPSI-MRI_CONTRA-WEIGHT-BLI_max_radiance | Day_nr-NS-BLI_max_flux | 0.8458 | 0.0305 |
| WEIGHT-NS-BLI_max_flux | Day_nr-MRI_IPSI-MRI_CONTRA-BLI_max_radiance | 0.8453 | 0.0764 |
| MRI_IPSI-WEIGHT-BLI_max_flux | Day_nr-MRI_CONTRA-NS-BLI_max_radiance | 0.8415 | 0.0495 |
| MRI_CONTRA-WEIGHT-BLI_max_radiance | Day_nr-MRI_IPSI-NS-BLI_max_flux | 0.8399 | 0.0404 |
| MRI_IPSI-MRI_CONTRA-WEIGHT-NS-BLI_max_radiance | Day_nr-BLI_max_flux | 0.8386 | 0.0349 |
| MRI_CONTRA-BLI_max_flux | Day_nr-MRI_IPSI-WEIGHT-NS-BLI_max_radiance | 0.8361 | 0.0261 |
| Day_nr-MRI_CONTRA-BLI_max_radiance | MRI_IPSI-WEIGHT-NS-BLI_max_flux | 0.8356 | 0.0319 |
| MRI_IPSI-BLI_max_radiance | Day_nr-MRI_CONTRA-WEIGHT-NS-BLI_max_flux | 0.8325 | 0.0316 |
| Day_nr-BLI_max_radiance | MRI_IPSI-MRI_CONTRA-WEIGHT-NS-BLI_max_flux | 0.8323 | 0.0269 |
| WEIGHT-BLI_max_flux | Day_nr-MRI_IPSI-MRI_CONTRA-NS-BLI_max_radiance | 0.8300 | 0.0452 |
| MRI_IPSI-WEIGHT-BLI_max_radiance | Day_nr-MRI_CONTRA-NS-BLI_max_flux | 0.8299 | 0.0374 |
| BLI_max_radiance | Day_nr-MRI_IPSI-MRI_CONTRA-WEIGHT-NS-BLI_max_flux | 0.8279 | 0.0258 |
| Day_nr-MRI_CONTRA- | MRI_IPSI-NS-BLI_max_flux | 0.8269 | 0.0517 |

| | | | |
|---|---|---|---|
| WEIGHT-BLI_max_radiance | | | |
| MRI_IPSI-MRI_CONTRA-BLI_max_radiance | Day_nr-WEIGHT-NS-BLI_max_flux | 0.8247 | 0.0246 |
| BLI_max_flux | Day_nr-MRI_IPSI-MRI_CONTRA-WEIGHT-NS-BLI_max_radiance | 0.8240 | 0.0273 |
| WEIGHT-BLI_max_radiance | Day_nr-MRI_IPSI-MRI_CONTRA-NS-BLI_max_flux | 0.8233 | 0.0389 |
| MRI_CONTRA-BLI_max_radiance | Day_nr-MRI_IPSI-WEIGHT-NS-BLI_max_flux | 0.8225 | 0.0307 |
| Day_nr-MRI_CONTRA | MRI_IPSI-WEIGHT-NS-BLI_max_flux-BLI_max_radiance | 0.8218 | 0.0243 |
| Day_nr-WEIGHT | MRI_IPSI-MRI_CONTRA-NS-BLI_max_flux-BLI_max_radiance | 0.8217 | 0.0292 |
| Day_nr-WEIGHT-BLI_max_radiance | MRI_IPSI-MRI_CONTRA-NS-BLI_max_flux | 0.8215 | 0.0351 |
| Day_nr-MRI_CONTRA-WEIGHT | MRI_IPSI-NS-BLI_max_flux-BLI_max_radiance | 0.8203 | 0.0461 |
| NS-BLI_max_radiance | Day_nr-MRI_IPSI-MRI_CONTRA-WEIGHT-BLI_max_flux | 0.8198 | 0.0302 |
| MRI_IPSI-BLI_max_flux | Day_nr-MRI_CONTRA-WEIGHT-NS-BLI_max_radiance | 0.8193 | 0.0286 |
| MRI_IPSI-MRI_CONTRA-BLI_max_flux | Day_nr-WEIGHT-NS-BLI_max_radiance | 0.8190 | 0.0264 |
| Day_nr | MRI_IPSI-MRI_CONTRA-WEIGHT-NS-BLI_max_flux-BLI_max_radiance | 0.8188 | 0.0196 |
| Day_nr-MRI_IPSI-MRI_CONTRA-WEIGHT | NS-BLI_max_flux-BLI_max_radiance | 0.8175 | 0.0276 |

| | | | |
|---|---|---|---|
| MRI_CONTRA-WEIGHT-NS-BLI_max_radiance | Day_nr-MRI_IPSI-BLI_max_flux | 0.8173 | 0.0523 |
| Day_nr-MRI_IPSI-MRI_CONTRA-WEIGHT-BLI_max_flux-BLI_max_radiance | NS | 0.8166 | 0.0681 |
| MRI_CONTRA-NS-BLI_max_radiance | Day_nr-MRI_IPSI-WEIGHT-BLI_max_flux | 0.8124 | 0.0253 |
| Day_nr-MRI_IPSI-WEIGHT | MRI_CONTRA-NS-BLI_max_flux-BLI_max_radiance | 0.8116 | 0.0312 |
| MRI_CONTRA-NS-BLI_max_flux | Day_nr-MRI_IPSI-WEIGHT-BLI_max_radiance | 0.8095 | 0.0425 |
| MRI_IPSI-NS-BLI_max_radiance | Day_nr-MRI_CONTRA-WEIGHT-BLI_max_flux | 0.8089 | 0.0351 |
| Day_nr-MRI_CONTRA-WEIGHT-BLI_max_flux | MRI_IPSI-NS-BLI_max_radiance | 0.8061 | 0.0375 |
| NS-BLI_max_flux | Day_nr-MRI_IPSI-MRI_CONTRA-WEIGHT-BLI_max_radiance | 0.8016 | 0.0387 |
| Day_nr-NS | MRI_IPSI-MRI_CONTRA-WEIGHT-BLI_max_flux-BLI_max_radiance | 0.8004 | 0.0336 |
| MRI_IPSI-MRI_CONTRA-NS-BLI_max_radiance | Day_nr-WEIGHT-BLI_max_flux | 0.7989 | 0.0286 |
| Day_nr-MRI_CONTRA-NS-BLI_max_radiance | MRI_IPSI-WEIGHT-BLI_max_flux | 0.7984 | 0.0277 |
| Day_nr-MRI_IPSI-MRI_CONTRA-WEIGHT-NS-BLI_max_radiance | BLI_max_flux | 0.7971 | 0.0400 |
| Day_nr-MRI_CONTRA-NS | MRI_IPSI-WEIGHT-BLI_max_flux-BLI_max_radiance | 0.7949 | 0.0254 |
| MRI_IPSI-WEIGHT-NS- | Day_nr-MRI_CONTRA- | 0.7935 | 0.0612 |

| | | | |
|---|---|---|---|
| BLI_max_flux | BLI_max_radiance | | |
| Day_nr-MRI_IPSI-MRI_CONTRA | WEIGHT-NS-BLI_max_flux-BLI_max_radiance | 0.7924 | 0.0303 |
| Day_nr-MRI_CONTRA-BLI_max_flux | MRI_IPSI-WEIGHT-NS-BLI_max_radiance | 0.7915 | 0.0321 |
| Day_nr-NS-BLI_max_radiance | MRI_IPSI-MRI_CONTRA-WEIGHT-BLI_max_flux | 0.7906 | 0.0440 |
| MRI_IPSI-MRI_CONTRA-NS-BLI_max_flux | Day_nr-WEIGHT-BLI_max_radiance | 0.7886 | 0.0412 |
| Day_nr-WEIGHT-BLI_max_flux | MRI_IPSI-MRI_CONTRA-NS-BLI_max_radiance | 0.7872 | 0.0345 |
| WEIGHT-NS-BLI_max_radiance | Day_nr-MRI_IPSI-MRI_CONTRA-BLI_max_flux | 0.7867 | 0.0363 |
| Day_nr-WEIGHT-NS | MRI_IPSI-MRI_CONTRA-BLI_max_flux-BLI_max_radiance | 0.7866 | 0.0275 |
| Day_nr-WEIGHT-NS-BLI_max_radiance | MRI_IPSI-MRI_CONTRA-BLI_max_flux | 0.7862 | 0.0314 |
| MRI_IPSI-WEIGHT-NS-BLI_max_radiance | Day_nr-MRI_CONTRA-BLI_max_flux | 0.7861 | 0.0388 |
| Day_nr-MRI_CONTRA-WEIGHT-NS | MRI_IPSI-BLI_max_flux-BLI_max_radiance | 0.7858 | 0.0481 |
| Day_nr-MRI_CONTRA-WEIGHT-BLI_max_flux-BLI_max_radiance | MRI_IPSI-NS | 0.7847 | 0.0400 |
| Day_nr-BLI_max_flux | MRI_IPSI-MRI_CONTRA-WEIGHT-NS-BLI_max_radiance | 0.7816 | 0.0438 |
| Day_nr-MRI_CONTRA-WEIGHT-NS-BLI_max_radiance | MRI_IPSI-BLI_max_flux | 0.7811 | 0.0471 |
| Day_nr-MRI_IPSI-MRI_CONTRA-NS | WEIGHT-BLI_max_flux-BLI_max_radiance | 0.7787 | 0.0251 |

| | | | |
|---|---|---|---|
| MRI_CONTRA-BLI_max_flux-BLI_max_radiance | Day_nr-MRI_IPSI-WEIGHT-NS | 0.7759 | 0.0323 |
| Day_nr-WEIGHT-BLI_max_flux-BLI_max_radiance | MRI_IPSI-MRI_CONTRA-NS | 0.7752 | 0.0542 |
| Day_nr-MRI_IPSI-MRI_CONTRA-WEIGHT-NS | BLI_max_flux-BLI_max_radiance | 0.7750 | 0.0304 |
| Day_nr-MRI_IPSI-MRI_CONTRA-WEIGHT-BLI_max_flux | NS-BLI_max_radiance | 0.7750 | 0.0384 |
| Day_nr-MRI_IPSI-WEIGHT-NS-BLI_max_radiance | MRI_CONTRA-BLI_max_flux | 0.7738 | 0.0356 |
| Day_nr-MRI_IPSI-WEIGHT-NS | MRI_CONTRA-BLI_max_flux-BLI_max_radiance | 0.7730 | 0.0398 |
| MRI_IPSI-NS-BLI_max_flux | Day_nr-MRI_CONTRA-WEIGHT-BLI_max_radiance | 0.7725 | 0.0461 |
| MRI_CONTRA-WEIGHT-BLI_max_flux-BLI_max_radiance | Day_nr-MRI_IPSI-NS | 0.7719 | 0.0450 |
| Day_nr-MRI_IPSI-WEIGHT-BLI_max_flux | MRI_CONTRA-NS-BLI_max_radiance | 0.7683 | 0.0371 |
| Day_nr-MRI_CONTRA-BLI_max_flux-BLI_max_radiance | MRI_IPSI-WEIGHT-NS | 0.7670 | 0.0405 |
| BLI_max_flux-BLI_max_radiance | Day_nr-MRI_IPSI-MRI_CONTRA-WEIGHT-NS | 0.7666 | 0.0327 |
| Day_nr-MRI_IPSI-MRI_CONTRA-NS-BLI_max_radiance | WEIGHT-BLI_max_flux | 0.7663 | 0.0694 |
| Day_nr-MRI_IPSI | MRI_CONTRA-WEIGHT-NS-BLI_max_flux-BLI_max_radiance | 0.7638 | 0.0406 |

| | | | |
|---|---|---|---|
| WEIGHT-BLI_max_flux-BLI_max_radiance | Day_nr-MRI_IPSI-MRI_CONTRA-NS | 0.7631 | 0.0311 |
| Day_nr-MRI_IPSI-MRI_CONTRA-BLI_max_flux | WEIGHT-NS-BLI_max_radiance | 0.7597 | 0.0356 |
| Day_nr-MRI_CONTRA-NS-BLI_max_flux | MRI_IPSI-WEIGHT-BLI_max_radiance | 0.7581 | 0.0282 |
| Day_nr-BLI_max_flux-BLI_max_radiance | MRI_IPSI-MRI_CONTRA-WEIGHT-NS | 0.7572 | 0.0333 |
| Day_nr-MRI_IPSI-NS | MRI_CONTRA-WEIGHT-BLI_max_flux-BLI_max_radiance | 0.7517 | 0.0389 |
| MRI_IPSI-MRI_CONTRA-WEIGHT-BLI_max_flux-BLI_max_radiance | Day_nr-NS | 0.7513 | 0.0402 |
| MRI_IPSI-MRI_CONTRA-BLI_max_flux-BLI_max_radiance | Day_nr-WEIGHT-NS | 0.7486 | 0.0353 |
| Day_nr-MRI_IPSI-BLI_max_radiance | MRI_CONTRA-WEIGHT-NS-BLI_max_flux | 0.7450 | 0.0449 |
| Day_nr-NS-BLI_max_flux | MRI_IPSI-MRI_CONTRA-WEIGHT-BLI_max_radiance | 0.7443 | 0.0388 |
| Day_nr-MRI_CONTRA-WEIGHT-NS-BLI_max_flux | MRI_IPSI-BLI_max_radiance | 0.7394 | 0.0518 |
| MRI_IPSI-BLI_max_flux-BLI_max_radiance | Day_nr-MRI_CONTRA-WEIGHT-NS | 0.7382 | 0.0232 |
| MRI_IPSI-WEIGHT-BLI_max_flux-BLI_max_radiance | Day_nr-MRI_CONTRA-NS | 0.7381 | 0.0321 |
| Day_nr-WEIGHT-NS-BLI_max_flux | MRI_IPSI-MRI_CONTRA-BLI_max_radiance | 0.7358 | 0.0386 |
| Day_nr-MRI_IPSI-MRI_CONTRA-WEIGHT-NS-BLI_max_flux | BLI_max_radiance | 0.7282 | 0.0353 |

| | | | |
|---|---|---|---|
| Day_nr-MRI_IPSI-BLI_max_flux | MRI_CONTRA-WEIGHT-NS-BLI_max_radiance | 0.7267 | 0.0268 |
| Day_nr-MRI_IPSI-NS-BLI_max_radiance | MRI_CONTRA-WEIGHT-BLI_max_flux | 0.7260 | 0.0335 |
| Day_nr-MRI_IPSI-WEIGHT-NS-BLI_max_flux | MRI_CONTRA-BLI_max_radiance | 0.7180 | 0.0830 |
| MRI_CONTRA-NS-BLI_max_flux-BLI_max_radiance | Day_nr-MRI_IPSI-WEIGHT | 0.7161 | 0.0317 |
| NS-BLI_max_flux-BLI_max_radiance | Day_nr-MRI_IPSI-MRI_CONTRA-WEIGHT | 0.7155 | 0.0277 |
| Day_nr-MRI_CONTRA-NS-BLI_max_flux-BLI_max_radiance | MRI_IPSI-WEIGHT | 0.7127 | 0.0237 |
| Day_nr-MRI_IPSI-NS-BLI_max_flux | MRI_CONTRA-WEIGHT-BLI_max_radiance | 0.7046 | 0.0260 |
| Day_nr-NS-BLI_max_flux-BLI_max_radiance | MRI_IPSI-MRI_CONTRA-WEIGHT | 0.6927 | 0.0272 |
| Day_nr-MRI_IPSI-BLI_max_flux-BLI_max_radiance | MRI_CONTRA-WEIGHT-NS | 0.6907 | 0.0202 |
| Day_nr-MRI_IPSI-MRI_CONTRA-NS-BLI_max_flux | WEIGHT-BLI_max_radiance | 0.6893 | 0.0387 |
| MRI_IPSI-NS-BLI_max_flux-BLI_max_radiance | Day_nr-MRI_CONTRA-WEIGHT | 0.6804 | 0.0258 |
| Day_nr-MRI_IPSI-NS-BLI_max_flux-BLI_max_radiance | MRI_CONTRA-WEIGHT | 0.6710 | 0.0193 |
| MRI_CONTRA-WEIGHT-NS-BLI_max_flux-BLI_max_radiance | Day_nr-MRI_IPSI | 0.6699 | 0.0396 |
| MRI_IPSI-MRI_CONTRA- | Day_nr-WEIGHT | 0.6631 | 0.0291 |

| | | | |
|---|---|---|---|
| NS-BLI_max_flux-BLI_max_radiance | | | |
| Day_nr-MRI_IPSI-MRI_CONTRA-BLI_max_flux-BLI_max_radiance | WEIGHT-NS | 0.6627 | 0.0386 |
| Day_nr-MRI_IPSI-WEIGHT-BLI_max_flux-BLI_max_radiance | MRI_CONTRA-NS | 0.6601 | 0.0407 |
| Day_nr-MRI_CONTRA-WEIGHT-NS-BLI_max_flux-BLI_max_radiance | MRI_IPSI | 0.6529 | 0.0627 |
| WEIGHT-NS-BLI_max_flux-BLI_max_radiance | Day_nr-MRI_IPSI-MRI_CONTRA | 0.6447 | 0.0431 |
| Day_nr-MRI_IPSI-MRI_CONTRA-NS-BLI_max_flux-BLI_max_radiance | WEIGHT | 0.6336 | 0.0233 |
| Day_nr-WEIGHT-NS-BLI_max_flux-BLI_max_radiance | MRI_IPSI-MRI_CONTRA | 0.6334 | 0.0415 |
| Day_nr-MRI_IPSI-WEIGHT-NS-BLI_max_flux-BLI_max_radiance | MRI_CONTRA | 0.5912 | 0.0228 |
| MRI_IPSI-WEIGHT-NS-BLI_max_flux-BLI_max_radiance | Day_nr-MRI_CONTRA | 0.5905 | 0.0226 |
| MRI_IPSI-MRI_CONTRA-WEIGHT-NS-BLI_max_flux-BLI_max_radiance | Day_nr | 0.4299 | 0.1477 |

ANN - artificial neural network; SD - standard deviation; MRI_CONTRA - volume of the contralateral hemisphere measured by MRI; MRI_IPSI - volume of the ipsilateral hemisphere measured by MRI; BLI_max_radiance - surface area of peak radiation measured by bioluminescence method; BLI_max_flux - surface area of peak growth measured by bioluminescence method; WEIGHT - animal weight; Day_nr - day from the middle carotid artery occlusion (MCAO) procedure; NS - scoring of phenotypic neurological assessment.
