# Supplementary material for: Innovative statistical approaches: the use of neural networks reduces the sample size in the splenectomy-MCAO mouse model
Source: Croat Med J. 2024 Apr;65(2):122–37. doi: 10.3325/cmj.2024.65.122 (PMC11074938; doi:10.3325/cmj.2024.65.122)
Supplement: Supplementary Table 7 [file CroatMedJ_65_s007.pdf]

Supplemental Table 7. Prediction accuracy of ANN class SPLX depending on the exclusion of variables and their combinations. The ANN was trained using a dataset with all days after a stroke, except the 2nd and 4th days. Values in the table are sorted based on the mean accuracy prediction of class SPLX.

"Out" variable	"In" variable	The mean accuracy value of ANN predictions for the SPLX class	SD of accuracy in predictions for the ANN SPLX class
MRI_IPSI-MRI_CONTRA-WEIGHT-NS	Day_nr-BLI_max_flux-BLI_max_radiance	0.9156	0.0303
MRI_IPSI-NS	Day_nr-MRI_CONTRA-WEIGHT-BLI_max_flux-BLI_max_radiance	0.9126	0.0267
MRI_IPSI-WEIGHT	Day_nr-MRI_CONTRA-NS-BLI_max_flux-BLI_max_radiance	0.9102	0.0352
MRI_IPSI-MRI_CONTRA-WEIGHT	Day_nr-NS-BLI_max_flux-BLI_max_radiance	0.9099	0.0310
MRI_IPSI	Day_nr-MRI_CONTRA-WEIGHT-NS-BLI_max_flux-BLI_max_radiance	0.9087	0.0361
NS	Day_nr-MRI_IPSI-MRI_CONTRA-WEIGHT-BLI_max_flux-BLI_max_radiance	0.9067	0.0268
MRI_IPSI-WEIGHT-NS	Day_nr-MRI_CONTRA-BLI_max_flux-BLI_max_radiance	0.9061	0.0240
MRI_CONTRA-WEIGHT-NS	Day_nr-MRI_IPSI-BLI_max_flux-BLI_max_radiance	0.9047	0.0277
MRI_IPSI-MRI_CONTRA-NS	Day_nr-WEIGHT-BLI_max_flux-	0.9039	0.0261

	BLI_max_radiance		
WEIGHT	Day_nr-MRI_IPSI-MRI_CONTRA-NS-BLI_max_flux-BLI_max_radiance	0.9021	0.0353
MRI_CONTRA-NS	Day_nr-MRI_IPSI-WEIGHT-BLI_max_flux-BLI_max_radiance	0.9019	0.0272
MRI_CONTRA-WEIGHT	Day_nr-MRI_IPSI-NS-BLI_max_flux-BLI_max_radiance	0.9000	0.0294
None	Day_nr-MRI_IPSI-MRI_CONTRA-WEIGHT-NS-BLI_max_flux-BLI_max_radiance	0.8993	0.0379
MRI_IPSI-MRI_CONTRA	Day_nr-WEIGHT-NS-BLI_max_flux-BLI_max_radiance	0.8993	0.0377
Day_nr-WEIGHT-NS	MRI_IPSI-MRI_CONTRA-BLI_max_flux-BLI_max_radiance	0.8942	0.0347
Day_nr-MRI_CONTRA-WEIGHT-NS	MRI_IPSI-BLI_max_flux-BLI_max_radiance	0.8942	0.0319
Day_nr-MRI_CONTRA-WEIGHT	MRI_IPSI-NS-BLI_max_flux-BLI_max_radiance	0.8940	0.0277
WEIGHT-NS	Day_nr-MRI_IPSI-MRI_CONTRA-BLI_max_flux-BLI_max_radiance	0.8938	0.0304
Day_nr-MRI_CONTRA	MRI_IPSI-WEIGHT-NS-BLI_max_flux-BLI_max_radiance	0.8925	0.0334
MRI_CONTRA	Day_nr-MRI_IPSI-WEIGHT-NS-BLI_max_flux-	0.8920	0.0421

	BLI_max_radiance		
Day_nr-MRI_CONTRA-NS	MRI_IPSI-WEIGHT-BLI_max_flux-BLI_max_radiance	0.8887	0.0362
MRI_IPSI-BLI_max_flux	Day_nr-MRI_CONTRA-WEIGHT-NS-BLI_max_radiance	0.8875	0.0241
Day_nr-WEIGHT	MRI_IPSI-MRI_CONTRA-NS-BLI_max_flux-BLI_max_radiance	0.8866	0.0343
Day_nr-MRI_IPSI-WEIGHT-NS	MRI_CONTRA-BLI_max_flux-BLI_max_radiance	0.8854	0.0335
Day_nr-MRI_IPSI-MRI_CONTRA	WEIGHT-NS-BLI_max_flux-BLI_max_radiance	0.8845	0.0322
MRI_IPSI-MRI_CONTRA-BLI_max_flux	Day_nr-WEIGHT-NS-BLI_max_radiance	0.8844	0.0278
Day_nr-MRI_IPSI-MRI_CONTRA-WEIGHT-NS	BLI_max_flux-BLI_max_radiance	0.8840	0.0296
MRI_IPSI-MRI_CONTRA-WEIGHT-BLI_max_flux	Day_nr-NS-BLI_max_radiance	0.8835	0.0250
BLI_max_flux	Day_nr-MRI_IPSI-MRI_CONTRA-WEIGHT-NS-BLI_max_radiance	0.8830	0.0317
Day_nr-NS	MRI_IPSI-MRI_CONTRA-WEIGHT-BLI_max_flux-BLI_max_radiance	0.8828	0.0275
Day_nr-MRI_IPSI-MRI_CONTRA-WEIGHT	NS-BLI_max_flux-BLI_max_radiance	0.8824	0.0369
Day_nr	MRI_IPSI-MRI_CONTRA-WEIGHT-NS-BLI_max_flux-BLI_max_radiance	0.8816	0.0375

MRI_CONTRA-BLI_max_flux	Day_nr-MRI_IPSI-WEIGHT-NS-BLI_max_radiance	0.8808	0.0302
Day_nr-MRI_IPSI	MRI_CONTRA-WEIGHT-NS-BLI_max_flux-BLI_max_radiance	0.8803	0.0323
WEIGHT-BLI_max_flux	Day_nr-MRI_IPSI-MRI_CONTRA-NS-BLI_max_radiance	0.8802	0.0332
MRI_IPSI-WEIGHT-BLI_max_flux	Day_nr-MRI_CONTRA-NS-BLI_max_radiance	0.8796	0.0366
Day_nr-MRI_IPSI-NS	MRI_CONTRA-WEIGHT-BLI_max_flux-BLI_max_radiance	0.8784	0.0287
Day_nr-MRI_IPSI-MRI_CONTRA-NS	WEIGHT-BLI_max_flux-BLI_max_radiance	0.8778	0.0330
MRI_CONTRA-WEIGHT-BLI_max_flux	Day_nr-MRI_IPSI-NS-BLI_max_radiance	0.8758	0.0314
Day_nr-BLI_max_flux	MRI_IPSI-MRI_CONTRA-WEIGHT-NS-BLI_max_radiance	0.8723	0.0289
Day_nr-MRI_CONTRA-BLI_max_flux	MRI_IPSI-WEIGHT-NS-BLI_max_radiance	0.8714	0.0342
Day_nr-WEIGHT-BLI_max_flux	MRI_IPSI-MRI_CONTRA-NS-BLI_max_radiance	0.8712	0.0328
Day_nr-MRI_IPSI-WEIGHT	MRI_CONTRA-NS-BLI_max_flux-BLI_max_radiance	0.8709	0.0353
Day_nr-MRI_CONTRA-WEIGHT-BLI_max_flux	MRI_IPSI-NS-BLI_max_radiance	0.8703	0.0366
MRI_CONTRA-NS-BLI_max_flux	Day_nr-MRI_IPSI-WEIGHT-BLI_max_radiance	0.8684	0.0342
Day_nr-MRI_IPSI-WEIGHT-	MRI_CONTRA-NS-	0.8676	0.0331

BLI_max_flux	BLI_max_radiance		
Day_nr-MRI_CONTRA-NS-BLI_max_flux	MRI_IPSI-WEIGHT-BLI_max_radiance	0.8667	0.0367
MRI_IPSI-MRI_CONTRA-NS-BLI_max_flux	Day_nr-WEIGHT-BLI_max_radiance	0.8658	0.0314
NS-BLI_max_flux	Day_nr-MRI_IPSI-MRI_CONTRA-WEIGHT-BLI_max_radiance	0.8639	0.0345
MRI_CONTRA-WEIGHT-NS-BLI_max_flux	Day_nr-MRI_IPSI-BLI_max_radiance	0.8638	0.0239
Day_nr-NS-BLI_max_flux	MRI_IPSI-MRI_CONTRA-WEIGHT-BLI_max_radiance	0.8635	0.0404
Day_nr-MRI_IPSI-MRI_CONTRA-NS-BLI_max_flux	WEIGHT-BLI_max_radiance	0.8616	0.0307
MRI_IPSI-NS-BLI_max_flux	Day_nr-MRI_CONTRA-WEIGHT-BLI_max_radiance	0.8613	0.0358
Day_nr-MRI_IPSI-BLI_max_flux	MRI_CONTRA-WEIGHT-NS-BLI_max_radiance	0.8580	0.0273
MRI_IPSI-MRI_CONTRA-WEIGHT-NS-BLI_max_flux	Day_nr-BLI_max_radiance	0.8578	0.0276
Day_nr-MRI_IPSI-MRI_CONTRA-WEIGHT-BLI_max_flux	NS-BLI_max_radiance	0.8564	0.0363
Day_nr-MRI_IPSI-MRI_CONTRA-BLI_max_flux	WEIGHT-NS-BLI_max_radiance	0.8563	0.0342
Day_nr-MRI_IPSI-NS-BLI_max_flux	MRI_CONTRA-WEIGHT-BLI_max_radiance	0.8563	0.0300
Day_nr-MRI_CONTRA-WEIGHT-NS-BLI_max_flux	MRI_IPSI-BLI_max_radiance	0.8561	0.0299
MRI_IPSI-BLI_max_radiance	Day_nr-MRI_CONTRA-	0.8547	0.0342

	WEIGHT-NS-BLI_max_flux		
MRI_IPSI-WEIGHT-NS-BLI_max_flux	Day_nr-MRI_CONTRA-BLI_max_radiance	0.8519	0.0400
Day_nr-WEIGHT-NS-BLI_max_flux	MRI_IPSI-MRI_CONTRA-BLI_max_radiance	0.8505	0.0321
WEIGHT-NS-BLI_max_flux	Day_nr-MRI_IPSI-MRI_CONTRA-BLI_max_radiance	0.8483	0.0341
BLI_max_radiance	Day_nr-MRI_IPSI-MRI_CONTRA-WEIGHT-NS-BLI_max_flux	0.8475	0.0325
Day_nr-MRI_IPSI-WEIGHT-NS-BLI_max_flux	MRI_CONTRA-BLI_max_radiance	0.8435	0.0305
MRI_IPSI-NS-BLI_max_radiance	Day_nr-MRI_CONTRA-WEIGHT-BLI_max_flux	0.8314	0.0350
NS-BLI_max_radiance	Day_nr-MRI_IPSI-MRI_CONTRA-WEIGHT-BLI_max_flux	0.8267	0.0298
Day_nr-MRI_IPSI-MRI_CONTRA-WEIGHT-NS-BLI_max_flux	BLI_max_radiance	0.8192	0.0293
MRI_CONTRA-BLI_max_radiance	Day_nr-MRI_IPSI-WEIGHT-NS-BLI_max_flux	0.8160	0.0363
WEIGHT-BLI_max_radiance	Day_nr-MRI_IPSI-MRI_CONTRA-NS-BLI_max_flux	0.8149	0.0331
MRI_IPSI-MRI_CONTRA-BLI_max_radiance	Day_nr-WEIGHT-NS-BLI_max_flux	0.8143	0.0398
MRI_IPSI-WEIGHT-BLI_max_radiance	Day_nr-MRI_CONTRA-NS-BLI_max_flux	0.8038	0.0435
MRI_IPSI-BLI_max_flux-BLI_max_radiance	Day_nr-MRI_CONTRA-WEIGHT-NS	0.7942	0.0375
BLI_max_flux-BLI_max_radiance	Day_nr-MRI_IPSI-MRI_CONTRA-WEIGHT-	0.7934	0.0360

	NS		
MRI_CONTRA-NS-BLI_max_radiance	Day_nr-MRI_IPSI-WEIGHT-BLI_max_flux	0.7934	0.0280
Day_nr-MRI_IPSI-BLI_max_radiance	MRI_CONTRA-WEIGHT-NS-BLI_max_flux	0.7932	0.0466
MRI_IPSI-MRI_CONTRA-NS-BLI_max_radiance	Day_nr-WEIGHT-BLI_max_flux	0.7924	0.0344
Day_nr-BLI_max_radiance	MRI_IPSI-MRI_CONTRA-WEIGHT-NS-BLI_max_flux	0.7882	0.0305
Day_nr-MRI_IPSI-BLI_max_flux-BLI_max_radiance	MRI_CONTRA-WEIGHT-NS	0.7850	0.0461
MRI_IPSI-WEIGHT-NS-BLI_max_radiance	Day_nr-MRI_CONTRA-BLI_max_flux	0.7816	0.0375
WEIGHT-NS-BLI_max_radiance	Day_nr-MRI_IPSI-MRI_CONTRA-BLI_max_flux	0.7798	0.0431
Day_nr-NS-BLI_max_radiance	MRI_IPSI-MRI_CONTRA-WEIGHT-BLI_max_flux	0.7786	0.0319
Day_nr-BLI_max_flux-BLI_max_radiance	MRI_IPSI-MRI_CONTRA-WEIGHT-NS	0.7765	0.0386
Day_nr-WEIGHT-BLI_max_radiance	MRI_IPSI-MRI_CONTRA-NS-BLI_max_flux	0.7610	0.0366
Day_nr-MRI_IPSI-NS-BLI_max_radiance	MRI_CONTRA-WEIGHT-BLI_max_flux	0.7593	0.0369
Day_nr-MRI_CONTRA-BLI_max_radiance	MRI_IPSI-WEIGHT-NS-BLI_max_flux	0.7556	0.0359
MRI_CONTRA-BLI_max_flux-BLI_max_radiance	Day_nr-MRI_IPSI-WEIGHT-NS	0.7550	0.0418
MRI_IPSI-MRI_CONTRA-WEIGHT-BLI_max_radiance	Day_nr-NS-BLI_max_flux	0.7530	0.0331
MRI_CONTRA-WEIGHT-	Day_nr-MRI_IPSI-NS-	0.7494	0.0372

BLI_max_radiance	BLI_max_flux		
MRI_IPSI-MRI_CONTRA-BLI_max_flux-BLI_max_radiance	Day_nr-WEIGHT-NS	0.7492	0.0391
Day_nr-WEIGHT-NS-BLI_max_radiance	MRI_IPSI-MRI_CONTRA-BLI_max_flux	0.7428	0.0358
Day_nr-MRI_IPSI-NS-BLI_max_flux-BLI_max_radiance	MRI_CONTRA-WEIGHT	0.7422	0.0428
Day_nr-MRI_CONTRA-NS-BLI_max_radiance	MRI_IPSI-WEIGHT-BLI_max_flux	0.7420	0.0347
Day_nr-NS-BLI_max_flux-BLI_max_radiance	MRI_IPSI-MRI_CONTRA-WEIGHT	0.7389	0.0355
MRI_IPSI-NS-BLI_max_flux-BLI_max_radiance	Day_nr-MRI_CONTRA-WEIGHT	0.7361	0.0384
MRI_IPSI-WEIGHT-BLI_max_flux-BLI_max_radiance	Day_nr-MRI_CONTRA-NS	0.7347	0.0527
NS-BLI_max_flux-BLI_max_radiance	Day_nr-MRI_IPSI-MRI_CONTRA-WEIGHT	0.7332	0.0314
Day_nr-MRI_IPSI-WEIGHT-NS-BLI_max_flux-BLI_max_radiance	MRI_CONTRA	0.7325	0.0433
WEIGHT-BLI_max_flux-BLI_max_radiance	Day_nr-MRI_IPSI-MRI_CONTRA-NS	0.7314	0.0474
Day_nr-MRI_CONTRA-BLI_max_flux-BLI_max_radiance	MRI_IPSI-WEIGHT-NS	0.7246	0.0399
MRI_IPSI-MRI_CONTRA-WEIGHT-NS-BLI_max_radiance	Day_nr-BLI_max_flux	0.7242	0.0346
Day_nr-WEIGHT-BLI_max_flux-BLI_max_radiance	MRI_IPSI-MRI_CONTRA-NS	0.7234	0.0398

MRI_CONTRA-WEIGHT-NS-BLI_max_radiance	Day_nr-MRI_IPSI-BLI_max_flux	0.7229	0.0355
MRI_IPSI-WEIGHT-NS-BLI_max_flux-BLI_max_radiance	Day_nr-MRI_CONTRA	0.7186	0.0485
Day_nr-MRI_IPSI-MRI_CONTRA-BLI_max_flux-BLI_max_radiance	WEIGHT-NS	0.7119	0.0512
Day_nr-MRI_IPSI-MRI_CONTRA-NS-BLI_max_flux-BLI_max_radiance	WEIGHT	0.7054	0.0421
Day_nr-MRI_CONTRA-WEIGHT-BLI_max_radiance	MRI_IPSI-NS-BLI_max_flux	0.7046	0.0427
Day_nr-WEIGHT-NS-BLI_max_flux-BLI_max_radiance	MRI_IPSI-MRI_CONTRA	0.7031	0.0558
Day_nr-MRI_IPSI-WEIGHT-BLI_max_flux-BLI_max_radiance	MRI_CONTRA-NS	0.7023	0.0473
MRI_CONTRA-NS-BLI_max_flux-BLI_max_radiance	Day_nr-MRI_IPSI-WEIGHT	0.6970	0.0369
WEIGHT-NS-BLI_max_flux-BLI_max_radiance	Day_nr-MRI_IPSI-MRI_CONTRA	0.6964	0.0523
MRI_IPSI-MRI_CONTRA-NS-BLI_max_flux-BLI_max_radiance	Day_nr-WEIGHT	0.6959	0.0329
Day_nr-MRI_IPSI-MRI_CONTRA-NS-BLI_max_radiance	WEIGHT-BLI_max_flux	0.6914	0.0448
Day_nr-MRI_IPSI-MRI_CONTRA-BLI_max_radiance	WEIGHT-NS-BLI_max_flux	0.6866	0.0542

Day_nr-MRI_CONTRA-NS-BLI_max_flux-BLI_max_radiance	MRI_IPSI-WEIGHT	0.6851	0.0446
Day_nr-MRI_IPSI-WEIGHT-BLI_max_radiance	MRI_CONTRA-NS-BLI_max_flux	0.6842	0.0508
Day_nr-MRI_CONTRA-WEIGHT-NS-BLI_max_radiance	MRI_IPSI-BLI_max_flux	0.6802	0.0425
Day_nr-MRI_IPSI-WEIGHT-NS-BLI_max_radiance	MRI_CONTRA-BLI_max_flux	0.6773	0.0515
MRI_CONTRA-WEIGHT-BLI_max_flux-BLI_max_radiance	Day_nr-MRI_IPSI-NS	0.6522	0.0473
Day_nr-MRI_IPSI-MRI_CONTRA-WEIGHT-BLI_max_radiance	NS-BLI_max_flux	0.6512	0.0376
MRI_IPSI-MRI_CONTRA-WEIGHT-BLI_max_flux-BLI_max_radiance	Day_nr-NS	0.6462	0.0396
Day_nr-MRI_IPSI-MRI_CONTRA-WEIGHT-NS-BLI_max_radiance	BLI_max_flux	0.6375	0.0372
Day_nr-MRI_CONTRA-WEIGHT-BLI_max_flux-BLI_max_radiance	MRI_IPSI-NS	0.6359	0.0474
Day_nr-MRI_IPSI-MRI_CONTRA-WEIGHT-BLI_max_flux-BLI_max_radiance	NS	0.6003	0.0356
Day_nr-MRI_CONTRA-WEIGHT-NS-BLI_max_flux-BLI_max_radiance	MRI_IPSI	0.5372	0.0391
MRI_CONTRA-WEIGHT-NS-BLI_max_flux-BLI_max_radiance	Day_nr-MRI_IPSI	0.5188	0.0334

MRI_IPSI-MRI_CONTRA-WEIGHT-NS-BLI_max_flux-BLI_max_radiance	Day_nr	0.3792	0.1951

ANN - artificial neural network; SPLX - splenectomized mice group; SD - standard deviation; MRI_CONTRA - volume of the contralateral hemisphere measured by MRI; MRI_IPSI - volume of the ipsilateral hemisphere measured by MRI; BLI_max_radiance - surface area of peak radiation measured by bioluminescence method; BLI_max_flux - surface area of peak growth measured by bioluminescence method; WEIGHT - animal weight; Day_nr - day from the middle carotid artery occlusion (MCAO) procedure; NS - scoring of phenotypic neurological assessment.
