# Supplementary material for: Innovative statistical approaches: the use of neural networks reduces the sample size in the splenectomy-MCAO mouse model
Source: Croat Med J. 2024 Apr;65(2):122–37. doi: 10.3325/cmj.2024.65.122 (PMC11074938; doi:10.3325/cmj.2024.65.122)
Supplement: Supplementary Table 8 [file CroatMedJ_65_s008.pdf]

Supplemental Table 8. Prediction accuracy of the ANN class SPL-sham depending on the exclusion of variables and their combinations. The ANN was trained using a dataset with all days after a stroke, except the 2nd and 4th days. Values in the table are sorted based on the mean accuracy prediction of class SPL-sham.

"Out" variable	"In" variable	The mean accuracy value of ANN predictions for the SPL-sham class	SD of accuracy in predictions for the ANN SPL-sham class
MRI_IPSI-WEIGHT	Day_nr-MRI_CONTRA-NS-BLI_max_flux-BLI_max_radiance	0.9867	0.0154
MRI_IPSI-MRI_CONTRA-WEIGHT	Day_nr-NS-BLI_max_flux-BLI_max_radiance	0.9840	0.0132
MRI_CONTRA-WEIGHT	Day_nr-MRI_IPSI-NS-BLI_max_flux-BLI_max_radiance	0.9838	0.0140
Day_nr-MRI_CONTRA-WEIGHT	MRI_IPSI-NS-BLI_max_flux-BLI_max_radiance	0.9809	0.0193
Day_nr-MRI_CONTRA-NS	MRI_IPSI-WEIGHT-BLI_max_flux-BLI_max_radiance	0.9807	0.0148
MRI_CONTRA-WEIGHT-NS	Day_nr-MRI_IPSI-BLI_max_flux-BLI_max_radiance	0.9802	0.0139
MRI_IPSI-NS	Day_nr-MRI_CONTRA-WEIGHT-BLI_max_flux-BLI_max_radiance	0.9801	0.0154
MRI_IPSI-WEIGHT-NS	Day_nr-MRI_CONTRA-BLI_max_flux-BLI_max_radiance	0.9789	0.0142
WEIGHT-NS	Day_nr-MRI_IPSI-MRI_CONTRA-BLI_max_flux-	0.9783	0.0211

	BLI_max_radiance		
MRI_IPSI-MRI_CONTRA-WEIGHT-NS	Day_nr-BLI_max_flux-BLI_max_radiance	0.9782	0.0146
MRI_CONTRA-NS	Day_nr-MRI_IPSI-WEIGHT-BLI_max_flux-BLI_max_radiance	0.9779	0.0136
WEIGHT	Day_nr-MRI_IPSI-MRI_CONTRA-NS-BLI_max_flux-BLI_max_radiance	0.9769	0.0250
MRI_IPSI-MRI_CONTRA-NS	Day_nr-WEIGHT-BLI_max_flux-BLI_max_radiance	0.9769	0.0147
MRI_IPSI-MRI_CONTRA	Day_nr-WEIGHT-NS-BLI_max_flux-BLI_max_radiance	0.9766	0.0163
MRI_CONTRA-WEIGHT-BLI_max_flux	Day_nr-MRI_IPSI-NS-BLI_max_radiance	0.9761	0.0149
Day_nr-MRI_CONTRA-WEIGHT-NS	MRI_IPSI-BLI_max_flux-BLI_max_radiance	0.9760	0.0189
MRI_CONTRA-WEIGHT-NS-BLI_max_flux	Day_nr-MRI_IPSI-BLI_max_radiance	0.9759	0.0225
MRI_IPSI	Day_nr-MRI_CONTRA-WEIGHT-NS-BLI_max_flux-BLI_max_radiance	0.9752	0.0195
Day_nr-MRI_CONTRA-WEIGHT-NS-BLI_max_flux	MRI_IPSI-BLI_max_radiance	0.9742	0.0251
MRI_CONTRA	Day_nr-MRI_IPSI-WEIGHT-NS-BLI_max_flux-BLI_max_radiance	0.9735	0.0198
NS	Day_nr-MRI_IPSI-MRI_CONTRA-WEIGHT-BLI_max_flux-BLI_max_radiance	0.9727	0.0183

MRI_IPSI-MRI_CONTRA-BLI_max_flux	Day_nr-WEIGHT-NS-BLI_max_radiance	0.9726	0.0124
MRI_IPSI-MRI_CONTRA-NS-BLI_max_flux	Day_nr-WEIGHT-BLI_max_radiance	0.9710	0.0198
MRI_IPSI-MRI_CONTRA-WEIGHT-BLI_max_flux	Day_nr-NS-BLI_max_radiance	0.9709	0.0165
MRI_IPSI-WEIGHT-NS-BLI_max_flux	Day_nr-MRI_CONTRA-BLI_max_radiance	0.9706	0.0222
Day_nr-WEIGHT	MRI_IPSI-MRI_CONTRA-NS-BLI_max_flux-BLI_max_radiance	0.9705	0.0200
MRI_CONTRA-BLI_max_flux	Day_nr-MRI_IPSI-WEIGHT-NS-BLI_max_radiance	0.9704	0.0154
None	Day_nr-MRI_IPSI-MRI_CONTRA-WEIGHT-NS-BLI_max_flux-BLI_max_radiance	0.9699	0.0223
MRI_CONTRA-NS-BLI_max_flux	Day_nr-MRI_IPSI-WEIGHT-BLI_max_radiance	0.9697	0.0155
Day_nr-NS	MRI_IPSI-MRI_CONTRA-WEIGHT-BLI_max_flux-BLI_max_radiance	0.9692	0.0179
Day_nr-MRI_CONTRA	MRI_IPSI-WEIGHT-NS-BLI_max_flux-BLI_max_radiance	0.9690	0.0216
Day_nr-MRI_CONTRA-WEIGHT-BLI_max_flux	MRI_IPSI-NS-BLI_max_radiance	0.9686	0.0269
Day_nr-MRI_IPSI-MRI_CONTRA-NS	WEIGHT-BLI_max_flux-BLI_max_radiance	0.9685	0.0249
MRI_IPSI-WEIGHT-BLI_max_flux	Day_nr-MRI_CONTRA-NS-BLI_max_radiance	0.9681	0.0176
Day_nr-WEIGHT-NS	MRI_IPSI-MRI_CONTRA-BLI_max_flux-BLI_max_radiance	0.9676	0.0162

Day_nr-MRI_CONTRA-NS-BLI_max_flux	MRI_IPSI-WEIGHT-BLI_max_radiance	0.9669	0.0217
WEIGHT-NS-BLI_max_flux	Day_nr-MRI_IPSI-MRI_CONTRA-BLI_max_radiance	0.9668	0.0216
WEIGHT-BLI_max_flux	Day_nr-MRI_IPSI-MRI_CONTRA-NS-BLI_max_radiance	0.9665	0.0213
Day_nr-MRI_CONTRA-BLI_max_flux	MRI_IPSI-WEIGHT-NS-BLI_max_radiance	0.9656	0.0169
MRI_IPSI-MRI_CONTRA-WEIGHT-NS-BLI_max_flux	Day_nr-BLI_max_radiance	0.9656	0.0240
Day_nr-MRI_IPSI-MRI_CONTRA-WEIGHT-NS	BLI_max_flux-BLI_max_radiance	0.9643	0.0239
MRI_IPSI-BLI_max_flux	Day_nr-MRI_CONTRA-WEIGHT-NS-BLI_max_radiance	0.9629	0.0179
Day_nr-WEIGHT-NS-BLI_max_flux	MRI_IPSI-MRI_CONTRA-BLI_max_radiance	0.9629	0.0259
Day_nr-WEIGHT-BLI_max_flux	MRI_IPSI-MRI_CONTRA-NS-BLI_max_radiance	0.9619	0.0232
BLI_max_flux	Day_nr-MRI_IPSI-MRI_CONTRA-WEIGHT-NS-BLI_max_radiance	0.9608	0.0200
Day_nr	MRI_IPSI-MRI_CONTRA-WEIGHT-NS-BLI_max_flux-BLI_max_radiance	0.9606	0.0185
Day_nr-MRI_IPSI-MRI_CONTRA	WEIGHT-NS-BLI_max_flux-BLI_max_radiance	0.9601	0.0176
MRI_IPSI-NS-BLI_max_flux	Day_nr-MRI_CONTRA-WEIGHT-BLI_max_radiance	0.9587	0.0296
Day_nr-MRI_IPSI-MRI_CONTRA-WEIGHT	NS-BLI_max_flux-BLI_max_radiance	0.9585	0.0244
Day_nr-MRI_IPSI-	BLI_max_radiance	0.9578	0.0237

MRI_CONTRA-WEIGHT-NS-BLI_max_flux			
Day_nr-MRI_IPSI-NS	MRI_CONTRA-WEIGHT-BLI_max_flux-BLI_max_radiance	0.9562	0.0267
Day_nr-MRI_IPSI-WEIGHT-NS	MRI_CONTRA-BLI_max_flux-BLI_max_radiance	0.9559	0.0232
Day_nr-MRI_IPSI-MRI_CONTRA-NS-BLI_max_flux	WEIGHT-BLI_max_radiance	0.9551	0.0200
NS-BLI_max_flux	Day_nr-MRI_IPSI-MRI_CONTRA-WEIGHT-BLI_max_radiance	0.9547	0.0305
Day_nr-NS-BLI_max_flux	MRI_IPSI-MRI_CONTRA-WEIGHT-BLI_max_radiance	0.9539	0.0256
Day_nr-BLI_max_flux	MRI_IPSI-MRI_CONTRA-WEIGHT-NS-BLI_max_radiance	0.9533	0.0235
Day_nr-MRI_IPSI-WEIGHT-BLI_max_flux	MRI_CONTRA-NS-BLI_max_radiance	0.9521	0.0212
Day_nr-MRI_IPSI-BLI_max_flux	MRI_CONTRA-WEIGHT-NS-BLI_max_radiance	0.9514	0.0287
Day_nr-MRI_IPSI-WEIGHT	MRI_CONTRA-NS-BLI_max_flux-BLI_max_radiance	0.9513	0.0215
Day_nr-MRI_IPSI-MRI_CONTRA-BLI_max_flux	WEIGHT-NS-BLI_max_radiance	0.9503	0.0288
Day_nr-MRI_IPSI-MRI_CONTRA-WEIGHT-BLI_max_flux	NS-BLI_max_radiance	0.9491	0.0281
Day_nr-MRI_IPSI	MRI_CONTRA-WEIGHT-NS-BLI_max_flux-BLI_max_radiance	0.9475	0.0262

Day_nr-MRI_IPSI-WEIGHT-NS-BLI_max_flux	MRI_CONTRA-BLI_max_radiance	0.9468	0.0285
Day_nr-MRI_IPSI-NS-BLI_max_flux	MRI_CONTRA-WEIGHT-BLI_max_radiance	0.9441	0.0311
Day_nr-MRI_IPSI-MRI_CONTRA-WEIGHT-NS-BLI_max_radiance	BLI_max_flux	0.8721	0.0481
MRI_IPSI-BLI_max_radiance	Day_nr-MRI_CONTRA-WEIGHT-NS-BLI_max_flux	0.8557	0.0314
MRI_IPSI-MRI_CONTRA-WEIGHT-BLI_max_radiance	Day_nr-NS-BLI_max_flux	0.8552	0.0407
Day_nr-MRI_IPSI-MRI_CONTRA-WEIGHT-BLI_max_radiance	NS-BLI_max_flux	0.8539	0.0481
MRI_IPSI-NS-BLI_max_radiance	Day_nr-MRI_CONTRA-WEIGHT-BLI_max_flux	0.8537	0.0337
MRI_CONTRA-WEIGHT-NS-BLI_max_radiance	Day_nr-MRI_IPSI-BLI_max_flux	0.8537	0.0455
Day_nr-MRI_CONTRA-WEIGHT-NS-BLI_max_radiance	MRI_IPSI-BLI_max_flux	0.8504	0.0600
MRI_IPSI-MRI_CONTRA-WEIGHT-NS-BLI_max_radiance	Day_nr-BLI_max_flux	0.8503	0.0691
NS-BLI_max_radiance	Day_nr-MRI_IPSI-MRI_CONTRA-WEIGHT-BLI_max_flux	0.8482	0.0328
BLI_max_radiance	Day_nr-MRI_IPSI-MRI_CONTRA-WEIGHT-NS-BLI_max_flux	0.8471	0.0340
MRI_IPSI-MRI_CONTRA-BLI_max_radiance	Day_nr-WEIGHT-NS-BLI_max_flux	0.8447	0.0337
MRI_IPSI-WEIGHT-NS-BLI_max_radiance	Day_nr-MRI_CONTRA-BLI_max_flux	0.8424	0.0472

MRI_IPSI-MRI_CONTRA-NS-BLI_max_radiance	Day_nr-WEIGHT-BLI_max_flux	0.8401	0.0358
MRI_CONTRA-WEIGHT-BLI_max_radiance	Day_nr-MRI_IPSI-NS-BLI_max_flux	0.8398	0.0464
MRI_CONTRA-NS-BLI_max_radiance	Day_nr-MRI_IPSI-WEIGHT-BLI_max_flux	0.8378	0.0372
MRI_IPSI-WEIGHT-BLI_max_radiance	Day_nr-MRI_CONTRA-NS-BLI_max_flux	0.8373	0.0381
WEIGHT-BLI_max_radiance	Day_nr-MRI_IPSI-MRI_CONTRA-NS-BLI_max_flux	0.8326	0.0303
MRI_CONTRA-BLI_max_radiance	Day_nr-MRI_IPSI-WEIGHT-NS-BLI_max_flux	0.8311	0.0387
WEIGHT-NS-BLI_max_radiance	Day_nr-MRI_IPSI-MRI_CONTRA-BLI_max_flux	0.8273	0.0509
Day_nr-BLI_max_radiance	MRI_IPSI-MRI_CONTRA-WEIGHT-NS-BLI_max_flux	0.8258	0.0430
Day_nr-MRI_CONTRA-BLI_max_radiance	MRI_IPSI-WEIGHT-NS-BLI_max_flux	0.8254	0.0503
Day_nr-MRI_CONTRA-WEIGHT-BLI_max_radiance	MRI_IPSI-NS-BLI_max_flux	0.8249	0.0632
Day_nr-MRI_CONTRA-NS-BLI_max_radiance	MRI_IPSI-WEIGHT-BLI_max_flux	0.8134	0.0471
Day_nr-NS-BLI_max_radiance	MRI_IPSI-MRI_CONTRA-WEIGHT-BLI_max_flux	0.8114	0.0400
Day_nr-WEIGHT-BLI_max_radiance	MRI_IPSI-MRI_CONTRA-NS-BLI_max_flux	0.8098	0.0472
Day_nr-WEIGHT-NS-BLI_max_radiance	MRI_IPSI-MRI_CONTRA-BLI_max_flux	0.8072	0.0407
Day_nr-MRI_IPSI-MRI_CONTRA-BLI_max_radiance	WEIGHT-NS-BLI_max_flux	0.7997	0.0756
Day_nr-MRI_IPSI-WEIGHT-	MRI_CONTRA-NS-	0.7978	0.0627

BLI_max_radiance	BLI_max_flux		
Day_nr-MRI_IPSI-WEIGHT-NS-BLI_max_radiance	MRI_CONTRA-BLI_max_flux	0.7975	0.0552
Day_nr-MRI_IPSI-MRI_CONTRA-NS-BLI_max_radiance	WEIGHT-BLI_max_flux	0.7945	0.0537
Day_nr-MRI_IPSI-BLI_max_radiance	MRI_CONTRA-WEIGHT-NS-BLI_max_flux	0.7808	0.0410
Day_nr-MRI_IPSI-NS-BLI_max_radiance	MRI_CONTRA-WEIGHT-BLI_max_flux	0.7744	0.0429
BLI_max_flux-BLI_max_radiance	Day_nr-MRI_IPSI-MRI_CONTRA-WEIGHT-NS	0.7651	0.0362
MRI_IPSI-BLI_max_flux-BLI_max_radiance	Day_nr-MRI_CONTRA-WEIGHT-NS	0.7614	0.0361
Day_nr-BLI_max_flux-BLI_max_radiance	MRI_IPSI-MRI_CONTRA-WEIGHT-NS	0.7461	0.0314
MRI_CONTRA-BLI_max_flux-BLI_max_radiance	Day_nr-MRI_IPSI-WEIGHT-NS	0.7404	0.0416
WEIGHT-BLI_max_flux-BLI_max_radiance	Day_nr-MRI_IPSI-MRI_CONTRA-NS	0.7343	0.0396
Day_nr-MRI_IPSI-BLI_max_flux-BLI_max_radiance	MRI_CONTRA-WEIGHT-NS	0.7312	0.0322
MRI_IPSI-MRI_CONTRA-BLI_max_flux-BLI_max_radiance	Day_nr-WEIGHT-NS	0.7304	0.0317
MRI_IPSI-WEIGHT-BLI_max_flux-BLI_max_radiance	Day_nr-MRI_CONTRA-NS	0.7258	0.0342
Day_nr-MRI_CONTRA-BLI_max_flux-BLI_max_radiance	MRI_IPSI-WEIGHT-NS	0.7252	0.0403
Day_nr-WEIGHT-	MRI_IPSI-MRI_CONTRA-NS	0.7172	0.0351

BLI_max_flux-BLI_max_radiance			
NS-BLI_max_flux-BLI_max_radiance	Day_nr-MRI_IPSI-MRI_CONTRA-WEIGHT	0.7155	0.0427
MRI_IPSI-NS-BLI_max_flux-BLI_max_radiance	Day_nr-MRI_CONTRA-WEIGHT	0.7086	0.0401
MRI_CONTRA-NS-BLI_max_flux-BLI_max_radiance	Day_nr-MRI_IPSI-WEIGHT	0.7060	0.0509
MRI_IPSI-MRI_CONTRA-WEIGHT-BLI_max_flux-BLI_max_radiance	Day_nr-NS	0.7049	0.0372
Day_nr-MRI_IPSI-MRI_CONTRA-WEIGHT-BLI_max_flux-BLI_max_radiance	NS	0.7007	0.0555
MRI_IPSI-MRI_CONTRA-NS-BLI_max_flux-BLI_max_radiance	Day_nr-WEIGHT	0.7003	0.0465
Day_nr-MRI_CONTRA-WEIGHT-BLI_max_flux-BLI_max_radiance	MRI_IPSI-NS	0.6970	0.0449
Day_nr-NS-BLI_max_flux-BLI_max_radiance	MRI_IPSI-MRI_CONTRA-WEIGHT	0.6876	0.0333
Day_nr-MRI_IPSI-NS-BLI_max_flux-BLI_max_radiance	MRI_CONTRA-WEIGHT	0.6874	0.0384
MRI_CONTRA-WEIGHT-BLI_max_flux-BLI_max_radiance	Day_nr-MRI_IPSI-NS	0.6873	0.0365
Day_nr-MRI_IPSI-MRI_CONTRA-BLI_max_flux-BLI_max_radiance	WEIGHT-NS	0.6859	0.0414
Day_nr-MRI_IPSI-WEIGHT-	MRI_CONTRA-NS	0.6822	0.0503

BLI_max_flux-BLI_max_radiance			
Day_nr-MRI_CONTRA-NS-BLI_max_flux-BLI_max_radiance	MRI_IPSI-WEIGHT	0.6796	0.0494
Day_nr-MRI_IPSI-MRI_CONTRA-NS-BLI_max_flux-BLI_max_radiance	WEIGHT	0.6525	0.0425
Day_nr-MRI_IPSI-WEIGHT-NS-BLI_max_flux-BLI_max_radiance	MRI_CONTRA	0.6011	0.0333
Day_nr-WEIGHT-NS-BLI_max_flux-BLI_max_radiance	MRI_IPSI-MRI_CONTRA	0.5994	0.0338
MRI_IPSI-WEIGHT-NS-BLI_max_flux-BLI_max_radiance	Day_nr-MRI_CONTRA	0.5946	0.0300
WEIGHT-NS-BLI_max_flux-BLI_max_radiance	Day_nr-MRI_IPSI-MRI_CONTRA	0.5918	0.0286
Day_nr-MRI_CONTRA-WEIGHT-NS-BLI_max_flux-BLI_max_radiance	MRI_IPSI	0.5576	0.0682
MRI_CONTRA-WEIGHT-NS-BLI_max_flux-BLI_max_radiance	Day_nr-MRI_IPSI	0.5313	0.0707
MRI_IPSI-MRI_CONTRA-WEIGHT-NS-BLI_max_flux-BLI_max_radiance	Day_nr	0.4190	0.1456

ANN - artificial neural network; SPL-sham - sham-operated mice group; SD - standard deviation; MRI_CONTRA - volume of the contralateral hemisphere measured by MRI; MRI_IPSI - volume of the ipsilateral hemisphere measured by MRI; BLI_max_radiance - surface area of peak radiation measured by bioluminescence method; BLI_max_flux - surface area of peak growth measured by bioluminescence method; WEIGHT - animal weight; Day_nr - day from the middle carotid artery occlusion (MCAO) procedure; NS - scoring of phenotypic neurological assessment
